# Immune Checkpoint Neuropilins as Novel Biomarkers and Therapeutic Targets for Pancreatic Cancer

**DOI:** 10.3390/cancers15082225

**Published:** 2023-04-10

**Authors:** Li-Hong He, Xiao-Zhen Zhang, Meng-Yi Lao, Han-Jia Zhang, Han-Shen Yang, Xue-Li Bai

**Affiliations:** 1 Department of Hepatobiliary and Pancreatic Surgery, The First Affiliated Hospital, School of Medicine, Zhejiang University, Hangzhou 310003, China; 22118032@zju.edu.cn (L.-H.H.);; 2 Zhejiang Provincial Key Laboratory of Pancreatic Disease, The First Affiliated Hospital, School of Medicine, Zhejiang University, Hangzhou 310009, China; 3 Zhejiang Provincial Innovation Center for the Study of Pancreatic Diseases, Hangzhou 310009, China; 4 Zhejiang Provincial Clinical Research Center for the Study of Hepatobiliary & Pancreatic Diseases, Hangzhou 310009, China; 5 Cancer Center, Zhejiang University, Hangzhou 310058, China

**Keywords:** neuropilins, immune checkpoint, tumor microenvironment, immunotherapy, pancreatic cancer

## Abstract

**Simple Summary:**

Malignant tumors, especially pancreatic cancer, are the major contributors to cancer mortality worldwide, and their effective treatments are still limited. Our study suggested that NRPs, especially NRP1, are attractive diagnostic and prognostic biomarkers, mediate immunoregulation, and have functions beyond immunology in the context of cancer. As potential novel immune checkpoints, NRPs, provide a new opportunity for tumor immunotherapy, and they will be of interest for further study.

**Abstract:**

The traditional immune checkpoint blockade therapy benefits some patients with cancer, but elicits no response in certain cancers, such as pancreatic adenocarcinoma (PAAD); thus, novel checkpoints and effective targets are required. Here, we found that there was a higher Neuropilin (NRP) expression in tumor tissues as novel immune checkpoints, which was associated with poor prognosis and pessimistic responses to immune checkpoint blockade therapy. In the tumor microenvironment of PAAD samples, NRPs were widely expressed in tumor, immune and stromal cells. The relationship of NRPs with tumor immunological features in PAAD and pan-cancer was evaluated using bioinformatics methods; it was positively correlated with the infiltration of myeloid immune cells and the expression of most immune checkpoint genes. Bioinformatics analysis, in vitro and in vivo experiments suggested that NRPs exhibit potential immune-related and immune-independent pro-tumor effects. NRPs, especially NRP1, are attractive biomarkers and therapeutic targets for cancers, particularly PAAD.

## 1. Introduction

Malignant tumors are a major contributor to the global burden of diseases, and their effective treatments are still limited [1,2]. Immunotherapy, especially immune checkpoint blockade (ICB) therapy, is considered one of the most promising therapies [3,4]. Although traditional ICBs, such as anti-PD1, -PD-L1, and -CTLA4, have shown considerable clinical benefits in some patients with cancers, including non-small cell lung cancer, melanoma, and colorectal cancer [4], there is one major challenge in the lack of response of certain cancers such as pancreatic adenocarcinoma (PAAD). Therefore, novel immune checkpoints and effective therapeutic targets need to be developed and evaluated.

Neuropilins (NRPs), which have two homologous isoforms (NRP1 and NRP2), are non-tyrosine kinase surface glycoproteins with a single-pass transmembrane, and are highly conserved across different species [5]. Both NRPs were initially found to be involved in semaphorin-mediated axon guidance as neuronal adhesion molecules and VEGF-mediated vascular biology, including embryonic vascular development (NRP1) and small lymphatic vessel and capillary formation (NRP2) [6]. Emerging studies have shown that NRPs have a variety of biological functions, including regulating immunity and tumorigenesis [6,7,8]. NRP1 has been defined as a novel immune checkpoint [9], and its blockade can enhance T-cell-mediated anti-tumor effects [10] and restore anti-tumor T-cell memory [11]. However, the potential functions and mechanisms of NRP1 and its homologous isoform, NRP2, in tumor progression and tumor immunology have not been fully elucidated.

In this study, we aim to thoroughly analyze the NRP-related immune landscape and biological functions of tumor immunology in PAAD and pan-cancer, to develop novel therapeutic targets. We investigated the expression characteristics of NRPs at the mRNA and protein levels, and the relationships between NRPs and prognosis, immune cell infiltration, immune checkpoint gene expression, and immunotherapy in pan-cancer, especially PAAD, using, but not limited to, bioinformatics. Furthermore, we conducted functional enrichment analysis and in vitro and in vivo experiments to elucidate the roles of NRPs in PAAD.

## 2. Materials and Methods

### 2.1. Data Source and Preprocessing

All RNA-Seq data and clinical information of 33 tumor samples from The Cancer Genome Atlas (TCGA) database (https://portal.gdc.cancer.gov/, accessed on 30 January 2022), and normal samples from the Genotype–Tissue Expression (GTEx) database (https://gtexportal.org/, accessed on 30 January 2022) were integrated and converted to the TPM format. Duplicate samples and cases with unavailable or unknown clinical features were removed. Statistical analysis and visualization were performed using the R software v3.6.3. The expression profile of NRP proteins in pan-cancer was obtained from the Human Protein Atlas (HPA) database (https://www.proteinatlas.org/, accessed on 30 January 2022). The expression profile of NRP mRNA from single-cell RNA-Seq in PAAD was obtained from the Tumor Immune Single-cell Hub (TISCH) database (http://tisch.comp-genomics.org/, accessed on 30 January 2022).

### 2.2. Collection of PAAD Patient Tissues

Paraffin-embedded and fresh human PAAD tissues and adjacent normal tissues were obtained from the Department of Hepatobiliary and Pancreatic Surgery, the First Affiliated Hospital, School of Medicine, Zhejiang University. All samples were pathologically confirmed. The fresh samples were used for experiments immediately or stored at −80 degrees. All protocols were approved by the Institutional Review Board of the First Affiliated Hospital, School of Medicine, Zhejiang University, and written informed consent was obtained from all patients at the time of enrollment. All experiments were reviewed and approved by the Ethics Committee of the First Affiliated Hospital, School of Medicine, Zhejiang University.

### 2.3. Western Blot, Immunohistochemistry, and Multiplexed Immunohistochemistry

For the Western blot, α-tubulin antibodies (1:2000 dilution, AF5012) served as internal controls and were purchased from Beyotime Biotechnology (Shanghai, China); NRP1 (1:5000 dilution, EPR3113) and NRP2 (1:1000 dilution, EPR23808-72) antibodies were purchased from Abcam (Cambridge, MA, USA) (for a detailed description, please refer to Supplementary Materials and Methods).

For immunohistochemistry, primary antibodies, NRP1 (ab81321, 1:300 dilution) and NRP2 (HPA039980, 1:200 dilution), were purchased from Abcam (Cambridge, MA, USA) and Atlas (Stockholm, Sweden), respectively (for a detailed description, please refer to Supplementary Materials and Methods).

The mIHC was performed by staining 4 μm thick formalin-fixed, paraffin-embedded whole tissue sections with standard primary antibodies sequentially and pairing them with the TSA 7-color kit (D110071-50T, Yuanxibio, Shanghai, China), then staining with DAPI. For example, deparaffinized slides were incubated with anti-panCK (AE1/AE3) antibody (#GM351507, Gene tech) for 60 min, and then treated with Alexa Fluor 488 goat anti-mouse IgG(H + L) peroxidase-conjugated (HRP) secondary antibody (#A10011-60, Yuanxibio, Shanghai, China) for 60 min. Then, DAPI was added for a nuclear stain, for 15 min, and the slides examined by scanning under a fluorescence microscope. The elution was completed after photographing. The second to sixth round of staining was then initiated, and the slides were washed in the TBST buffer and then transferred to a preheated EDTA solution (100 °C), before being heat-treated using a microwave set at 20% of the maximum power for 15 min. Slides were cooled in the same solution to room temperature, incubated with anti-CD8 (#BX50036-C3) for 60 min and then treated with peroxidase-conjugated (HRP) secondary antibody (#DS9800, Leica, Hesse, Germany) for 10 min. Then, labelling was developed for a strictly observed 10 min, using TSA 620, per manufacturer’s direction. Between all steps, the slides were washed with the Tris-buffer. The same process was repeated for the antibodies/fluorescent dyes, in the following order: anti-CD68 (#BX50031, Biolynx, Hangzhou, China)/TSA 520, anti-a-SMA (#19245s, CST, Danvers, MA, USA)/TSA 670, anti-CD4 (#ab133616, abcam, Cambridge, UK)/TSA 570, anti-NRP1 (#ab81321, abcam)/TSA 440 and anti-NRP2 (#HPA039980, Atlas, Stockholm, Sweden)/TSA 440. Each slide was then treated with 2 drops of DAPI (D1306; Thermofisher, Shanghai, China), washed in distilled water, and manually cover-slipped. Slides were air-dried, and pictures were taken with Pannoramic MIDI tissue imaging system (3DHISTECH). Images were analyzed using the Indica Halo software v3.1 (lndica Labs, Marlborough, MA, USA).

### 2.4. Tumor Immunology

The correlation between the expression of NRPs and the level of immune cell infiltration was quantified separately using ssGSEA (GSVA R package v.1.34.0) [12], MCP-counter and QuanTIseq (Immunedeconv R package) algorithms. The immune, stromal and estimated scores were investigated by estimating the stromal and immune cells in malignant tumor tissues, using expression data (ESTIMATE) algorithms (ESTIMATE R package). A list of immune cell infiltration signature genes used in the ssGSEA was obtained from previously published studies [13,14]. The correlation between NRPs with a list of immune checkpoint genes in various cancer types was explored using the Gene Corr module from the TIMER2.0 (http://timer.cistrome.org/, accessed on 30 January 2022) database [15]. Raw counts of RNA-seq data (level 3) and the corresponding clinical information were obtained from the TCGA-PAAD cohort, and the potential ICB response was predicted using the tumor immune dysfunction and exclusion (TIDE) algorithm [16].

### 2.5. Differentially Expressed Gene (DEG) Analysis and Functional Enrichment

In total, 178 patients with PAAD were separated into NRP^high^ and NRP^low^ expression groups according to the NRP median value. The DESeq2 R package (v.1.26.0) [17] was used to identify DEGs between the groups, where “Adjusted *p* < 0.05 and | Log_2_^(Fold Change)^ | >2” was set as a threshold. The ggplot2 R package (v.3.3.3) was used to present the volcano and heatmap plots. The ClusterProfiler R package (v.3.14.3) [18] was used to analyze the Gene Ontology (GO) functions of NRP-related DEGs and enriched Kyoto Encyclopedia of Gene and Genome (KEGG) pathways. Based on the reference gene sets (C2: curated gene sets; H: hallmark gene sets; C5: ontology gene sets) from MSigDB [19], we performed a gene set enrichment analysis (GSEA) functional enrichment analysis of the DEGs, and a false discovery rate of <0.25 and an adjusted *p*-value of <0.05 were identified as significant parameters.

### 2.6. In Vitro and In Vivo Experiments

The NRP1 knockdown PANC-1 cell line and NRP2 knockdown CFPAC-1 cell line were constructed with shNRP1 and shNRP2 plasmids, respectively. In vitro human pancreatic cancer cell proliferation, invasion, and migration were examined using the CCK8, Transwell, and scratch wound-healing assays, respectively. To investigate the effects of NRP1 inhibitor on the growth of PAAD in vivo, KPC cells (8 × 10^5^/25 μL, a mouse pancreatic cancer cell line) with the Matrigel matrix (Corning, NY, USA) were inoculated into the orthotopic pancreas of nude mice (immunodeficient) and C57BL/6 mice (immunocompetent) separately, and the NRP1 inhibitor, EG01377 (10 mg/kg/day; MCE, Monmouth Junction, NJ, USA), and an equal amount of vehicle DMSO were intraperitoneally injected after 5 days. The weight of tumors was recorded 2–3 weeks after injection. The same operation was used to investigate whether the NRP1 inhibitor affected the survival time of tumor-bearing mice, and the time of death of each mouse was tracked and recorded since the drug intervention. See Supplementary Materials and Methods for further details.

### 2.7. Statistical Analysis

Data were analyzed using the GraphPad Prism v9 software (GraphPad Software Inc., California, USA). Quantitative data from at least three replicates were presented as the mean ± standard error of the mean, and evaluated by an independent sample t-test if they followed a normal distribution. Correlations of NRP expression and traditional immune checkpoints were calculated using Spearman’s rho correlation coefficients, and heatmaps gave the purity-adjusted partial Spearman’s rho value as the degree of their correlation. Statistical difference was significant when *p* < 0.05.

## 3. Results

### 3.1. NRP Expression Analysis in PAAD and Other Cancers

**Figure 1:**
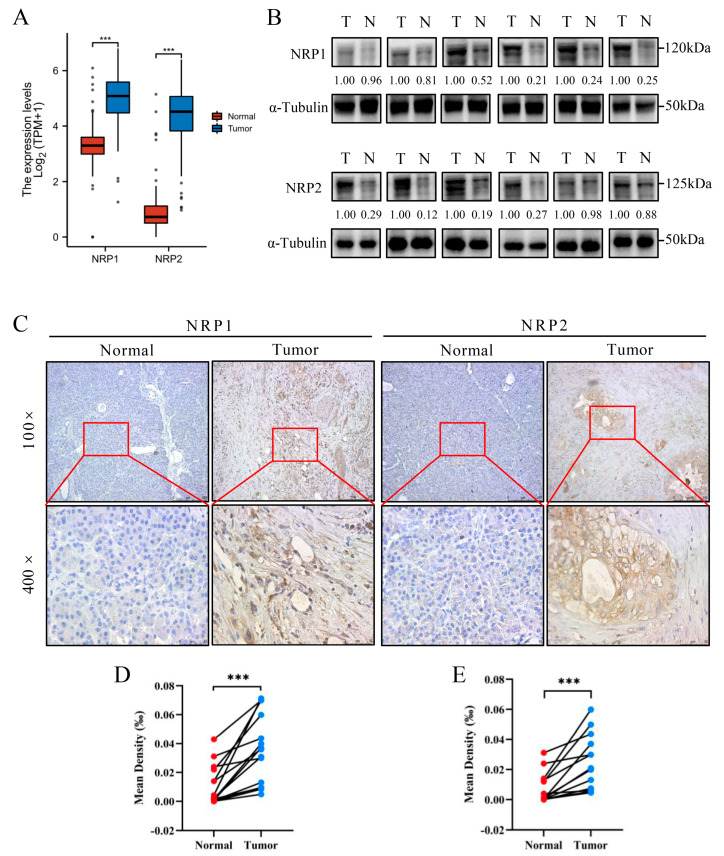
NRP expression in tumor tissues and normal tissues. (**A**) NRP mRNA expression between PAAD tumor tissues (179 PAAD patients) from the TCGA database and normal pancreas tissues from the TCGA (adjacent normal tissues from four PAAD patients) and GTEx databases (167 healthy people). (**B**) Western blot for NRP protein expression in fresh tumor tissues (T) and paracarcinoma (normal, N) tissues from six PAAD patients. (**C**–**E**) Immunohistochemistry (paraffin sections) for NRP protein expression in tumor tissues (T) and paracarcinoma (normal, N) tissues from 15 PAAD patients. *** *p* < 0.001. The uncropped blots are shown in Figure S8.

First, we analyzed the differences in the expression of NRPs in PAAD tumor samples and normal pancreas samples, and the results showed that both, NRP1 and NRP2, were significantly more expressed in PAAD tumor samples (*p* < 0.001, Figure 1A), and 17 other cancer types had similar results (Supplementary Figure S1A). Second, the differential expression of NRPs at the protein level was explored via Western blotting (Figure 1B) and immunohistochemistry (Figure 1C–E), which suggested that the expression of NRP proteins in PAAD tumor tissues (T) was higher than that in the adjacent normal tissues (N). In the HPA database, the expression of NRP1 was characterized by weak-to-moderate cytoplasmic positivity, often with a granular pattern in most cancer tissues, but this was negative in carcinoids and several cases of skin and cervical cancers (Supplementary Figure S1B). NRP2 was characterized by skin cancer, several urothelial cancers, and a few lung cancers, showing moderate-to-strong cytoplasmic and/or membranous positivity; however, cervical cancers, along with several colorectal, gastric, pancreatic and liver cancers, showed moderate cytoplasmic and/or membranous immunoreactivity with additional nuclear membranous staining in several cases (Supplementary Figure S1B).

**Figure 2:**
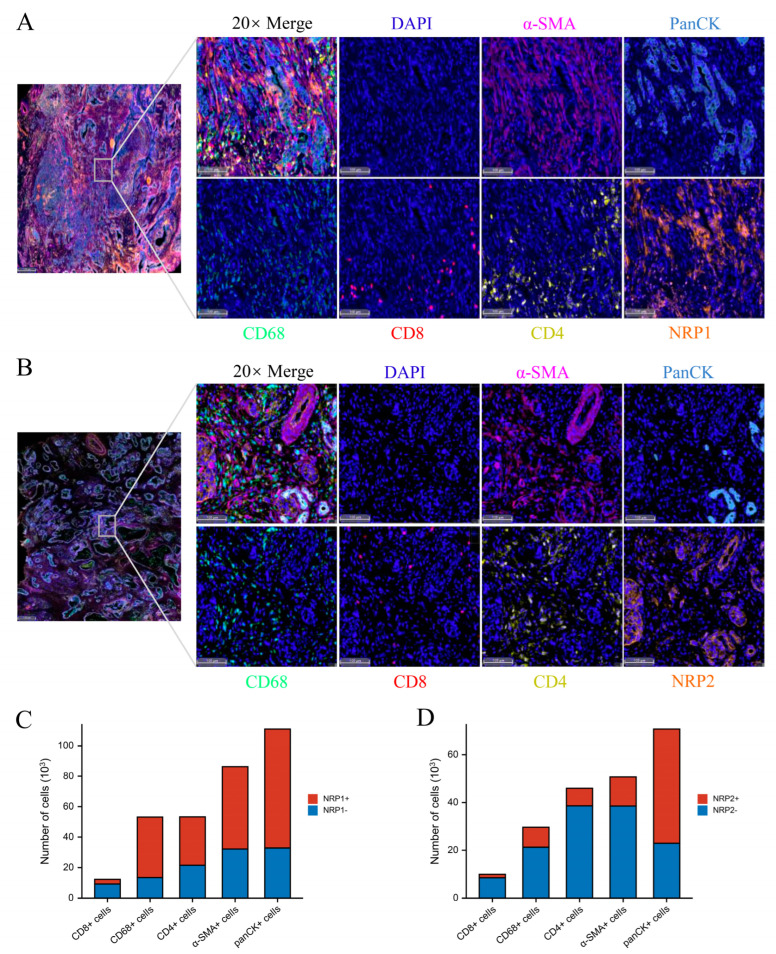
NRP expression in PAAD tumor microenvironment. (**A**,**B**) The representative results of multiplexed immunohistochemistry in PAAD tumor microenvironment. scale bars 100 μm. (**C**,**D**) The statistical results of NRP protein expression in various cells.

Finally, to evaluate the expression of NRPs in the tumor microenvironment (TME), we conducted an mIHC experiment on human PAAD tumor samples, which showed that NRP1 and NRP2 were widely expressed in numerous cell types, including tumor cells (panCK^+^), cancer-associated fibroblasts (CAF, α-SMA^+^), tumor-associated macrophages (TAM, CD68^+^), CD8^+^ T cells (CD8^+^) and CD4^+^ T cells (CD4^+^) (Figure 2A,B), and the specific statistics are shown in Figure 2C,D. Moreover, the Tumor Immune Single-cell Hub (TISCH) database was used to evaluate the expression of NRPs in the TME, from the perspective of single-cell RNA sequencing, which also showed that NRPs were expressed to varying degrees in tumor cells, immune cells, and stromal cells (Supplementary Figure S3).

### 3.2. NRPs Are Valuable Diagnostic and Prognostic Biomarkers

**Figure 3:**
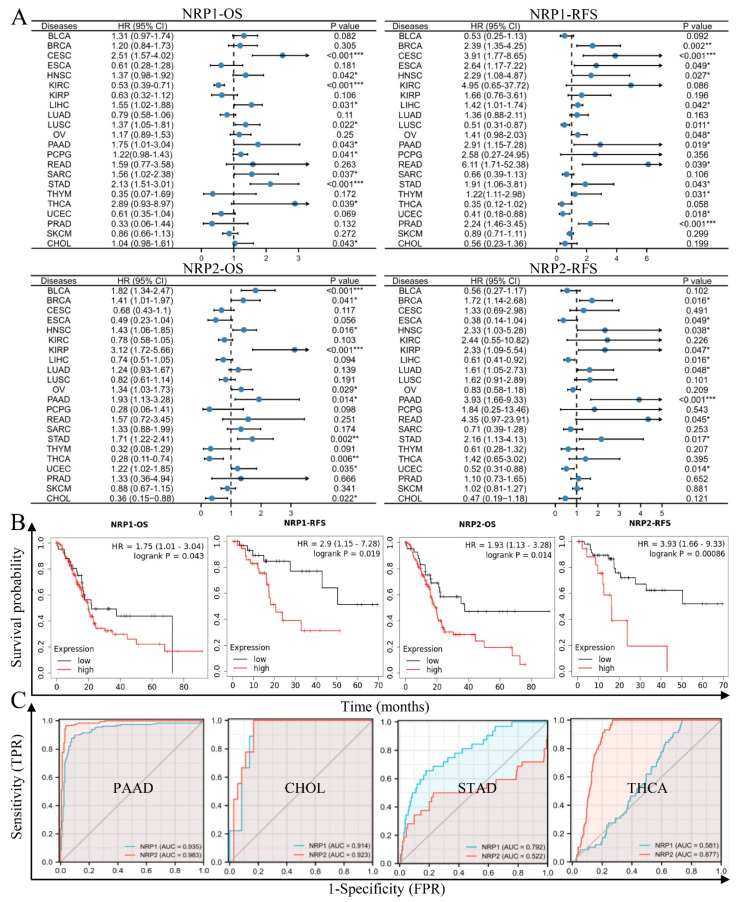
The prognostic and diagnostic significance of NRPs. (**A**,**B**) Log-rank test was used to analyze the relationship between NRP expression and OS, as well as RFS in pan-cancer, and the results are presented as forest plots (**A**); in PAAD, the results are presented as Kaplan–Meier plots (**B**). (**C**) Receiver operating characteristic (ROC) curve analysis evaluating the performance of NRPs for PAAD, CHOL, STAD and THCA diagnosis. The median value of NRP expression was taken as the cut-off value, and the patients were grouped into high- or low-expression groups. * *p* < 0.05; ** *p* < 0.01 and *** *p* < 0.001.

We further investigated the prognostic significance of NRPs in patients with cancer. In the log-rank test, the overall survival (OS) results revealed that NRP1 acts as a risk factor for patients with CSEC, LIHC, LUSC, PAAD, SARC and STAD, and as a protective factor in patients with KIRC. NRP2 acts as a risk factor for patients with BLCA, BRCA, HNSC, KIRP, OV, PAAD, STAD and UCEC, and as a protective factor for patients with THCA and CHOL. Moreover, the recurrence-free survival (RFS) results revealed that NRP1 acts as a risk factor for patients with BRCA, CESC, ESCA, HNSC, LIHC, PAAD, READ, STAD, THYM and PRAD, and as a protective factor for patients with LUSC and UCEC. NRP2 acts as a risk factor for patients with BRCA, HNSC, KIRP, LUAD, PAAD and STAD, and as a protective factor for patients with LIHC and UCEC (Figure 3A). It is noteworthy that the high expression of NRP1 and NRP2 was associated with a poor prognosis in PAAD in terms of OS or RFS, and this result was obtained using the Kaplan–Meier method (Figure 3B). Next, we estimated the diagnostic performance of NRPs using receiver operating characteristic (ROC) curves. As expected, NRPs showed significantly higher sensitivity and specificity for the diagnosis of various cancers, especially PAAD, and NRP1 also showed a high diagnostic value in CHOL. This result is consistent with our previous research results [20] (Figure 3C, Supplementary Figure S2).

### 3.3. Associations of the NRP Family and Immune Infiltration

**Figure 4:**
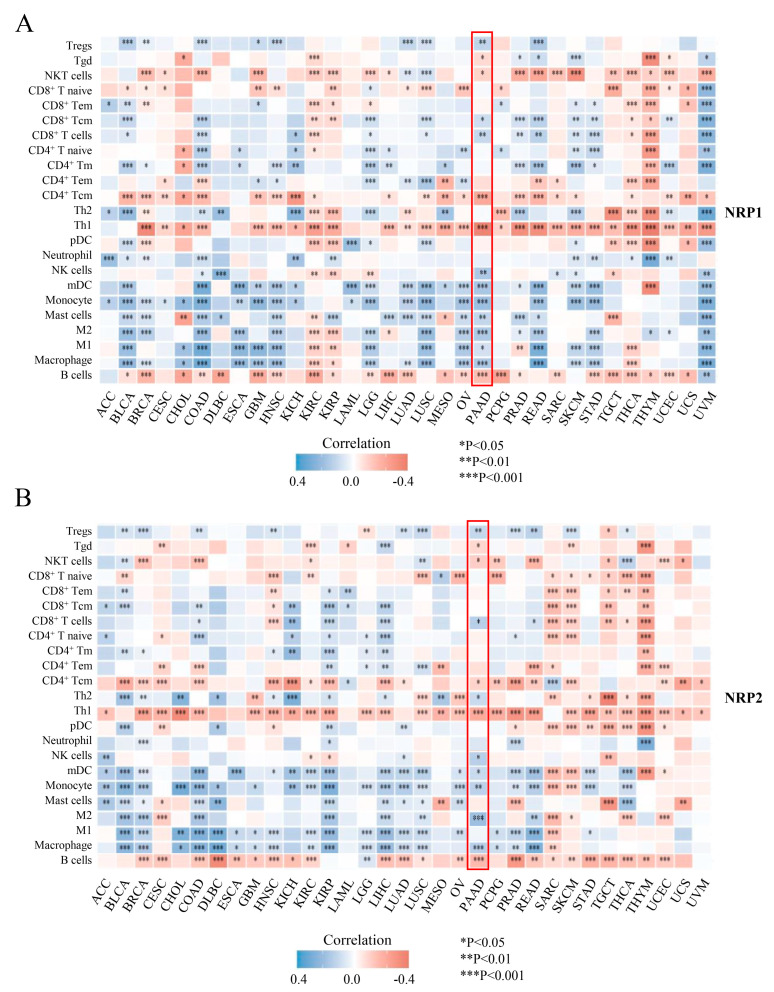
Tumor-immunology analysis. The correlation between NRP1 (**A**), as well as NRP2 (**B**), and the immune cell infiltration level using ssGSEA. The results of the PAAD are highlighted in the “red box”. * *p* < 0.05; ** *p* < 0.01 and *** *p* < 0.001.

Recent studies have shown that NRPs are expressed in various subsets of immune cells and are important for regulating immune responses [10,11,21,22,23]. To further explore the role of NRPs in tumor immunology, we determined the correlation between NRP expression and immune cell infiltration in PAAD and pan-cancer. In the ssGSEA algorithm (Figure 4), the expression of NRPs was significantly correlated with a variety of immune cell infiltrations in pan-cancer. Notably, the expression of NRPs was positively correlated with the infiltration of myeloid immune cells (e.g., monocytes, macrophages, mast cells and myeloid dendritic cells) in nearly all cancers, including PAAD, suggesting that NRPs play a vital role in the regulation of innate immunity. For lymphoid immune cells, the expression of NRP1 was negatively correlated with the infiltration of B and T helper (Th) 1 cells, CD4^+^T central memory (Tcm), and NKT cells in almost all cancers, and NRP2 presented similar results. These lymphocytes may also be the main mediators of NRP-regulated tumor immunity.

PAAD has an intrinsically complex TME. To verify and supplement the above results, we further investigated the correlation between NRP expression and immune cell infiltration in PAAD using two other algorithms, MCP-counter (Supplementary Figure S4A,B) and QuantTIseq (Supplementary Figure S4C,D) [24]. These chordal results showed a positive correlation between the expression of NRP and infiltration of macrophages, natural killer (NK) cells, dendritic cells (DCs), CAF, regulatory T (Treg) cells and CD8^+^ T cells (R > 0.2, *p* < 0.05), but negatively correlated with the infiltration of CD4^+^ Tcm cells (R < −0.2, *p* < 0.05). Interestingly, the infiltration of type 1 macrophages (M1), CD8^+^ T central memory (Tcm) cells and mast cells (R = −0.288, *p* = 0.031) was only significantly correlated with NRP1 expression (Figure 4A, Supplementary Figure S4A,C). In comparison, the infiltration of Th2 cells was only significantly correlated with NRP2 expression (Figure 4B, Supplementary Figure S4B,D). Given the important influence of non-tumor components on PAAD, we also investigated the total abundance of immune and stromal cells in individual PAAD samples using the ESTIMATE method, and found that the immune, stromal, and estimated scores were higher in the high NRP1 expression group (*p* < 0.001, Supplementary Figure S4E), but NRP2 expression was only significantly correlated with the stromal score (*p* < 0.05, Supplementary Figure S4F). These findings suggest that the NRP family has a complex effect on the immune microenvironment of PAAD, from innate to adaptive immunity, shifting the balance between immunosuppression and activation.

### 3.4. Association between NRPs and Immune Checkpoints

**Figure 5:**
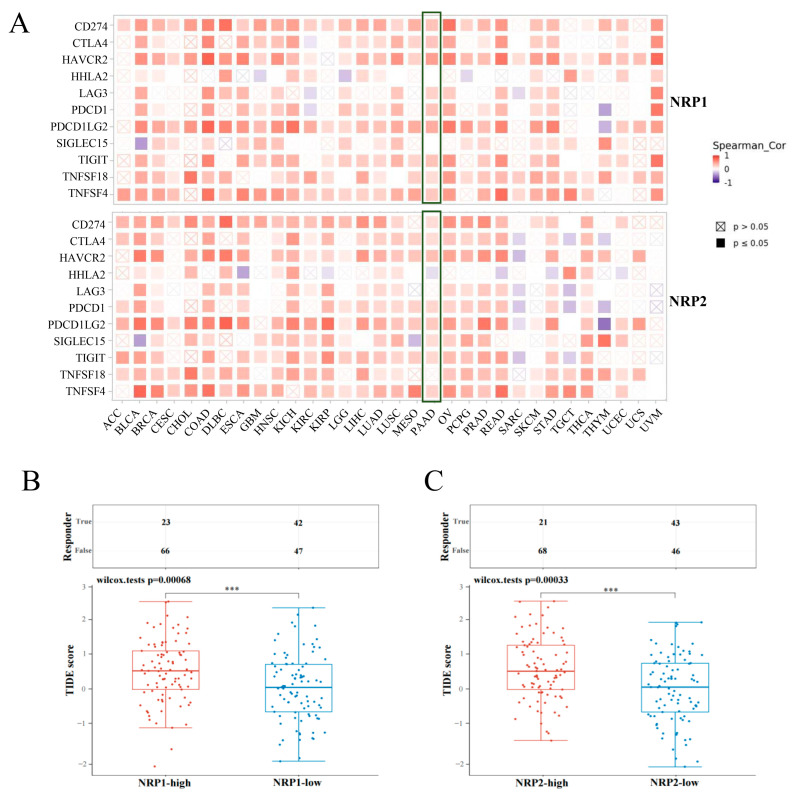
Tumor immunology analysis. (**A**) The heatmaps represent the correlation between NRP expression and immune check point expression in pan-cancer. Solid black square, *p* ≤ 0.05; hollow block with cross, *p* > 0.05; The “large green box” data belongs to PAAD. The correlation between NRP1 (**B**), as well as NRP2 (**C**), and ICB response prediction, and statistical tables of the ICB response and TIDE score in NRP high and low groups in PAAD were conducted. The higher the TIDE score, the poorer the efficacy of ICB, and the survival time after ICB treatment was short. *** *p* < 0.001.

Of further interest is whether NRP expression is associated with traditional immune checkpoints. We collected 11 common immune checkpoint genes, the correlation of which was assessed, with NRP expression, using the TIMER2.0 database [15]. In nearly all cancers, NRP1 expression was positively correlated with the expression of most immune checkpoint genes. The results in the NRP2 group were similar to those in the NRP1 group, but in SARC and UVM, NRP2 expression was negatively correlated and non-statistically correlated, respectively, with the expression of most immune checkpoint genes (Figure 5A). Figure S5 shows the details of the correlation between NRP expression and immune checkpoint gene expression in PAAD. These results suggest that NRP1 and NRP2 are involved in tumor immune evasion by interacting with immune checkpoints.

### 3.5. Association of NRPs and Tumor Immunotherapy

ICB, the most important tumor immunotherapy, has been shown to considerably improve antitumor efficacy, but the response of pancreatic cancer to ICB is not promising. Here, we predicted the response of different NRP expression levels to immune checkpoint inhibitors in PAAD using the TIDE algorithm, a tool to evaluate the dysfunction of tumor-infiltrating cytotoxic T lymphocytes and the rejection of it by immunosuppressive factors based on gene expression [16]. Interestingly, the high NRP1 and NRP2 expression groups had higher tide scores, which means that the curative effect of ICB was poor and the survival time after ICB treatment was short (Figure 5B,C). These findings highlight the possibility that NRP1 and NRP2 can predict the efficacy of tumor immunotherapy.

### 3.6. In Vitro and In Vivo Experiments and Functional Enrichment of NRPs in PAAD

**Figure 6:**
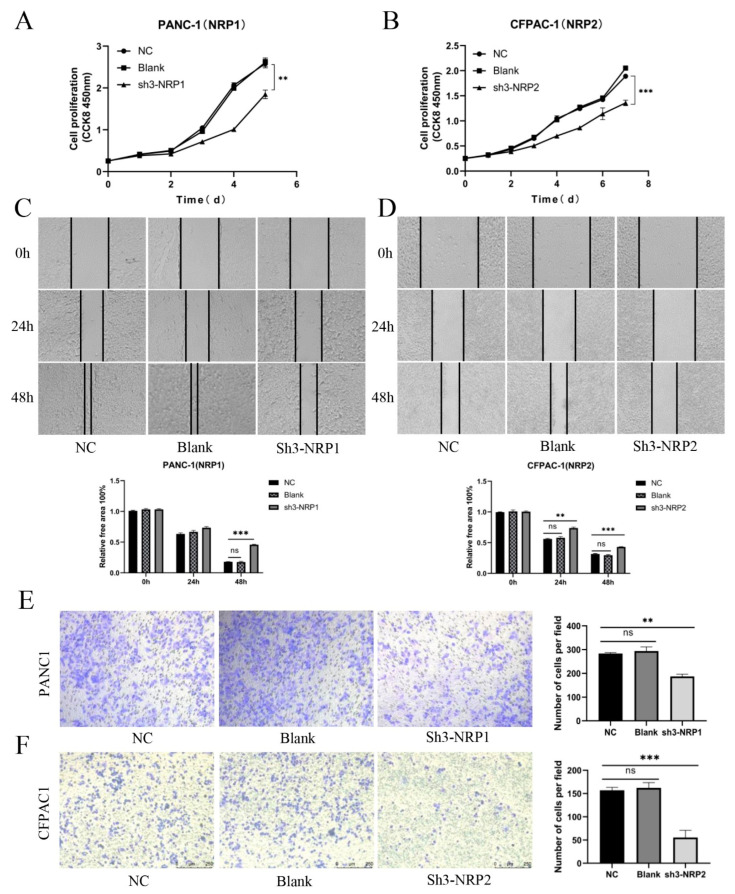
In vitro experiments. (**A**,**B**) CCK8 assay for the proliferation of PAAD cells after NRP gene silencing. (**C**,**D**) Wound-healing assay for the migration of PAAD cells after NRP gene silencing. Magnification 50×. (**E**,**F**) Transwell assay for the invasion of PAAD cells after NRP gene silencing. NC, negative control; Blank, not transfected. Magnification 50×. All experiments take the NC group as the reference. ** *p* < 0.01 and *** *p* < 0.001; ns, not significant.

**Figure 7:**
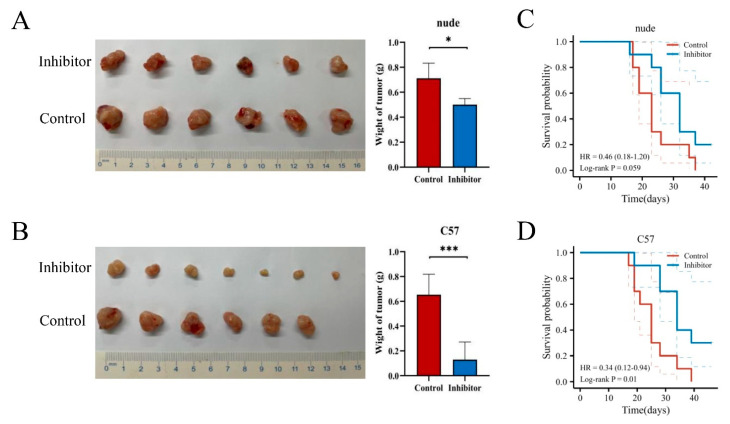
In vivo experiments. The visual maps of tumors, average tumor and tumor weight of the (**A**) immunodeficient nude mice (n = 6 per group) and (**B**) immunocompetent C57BL/6 mice (n = 7 per group). The growth curve of the (**C**) immunodeficient nude mice (n = 10 per group) and (**D**) immunocompetent C57BL/6 mice (n = 10 per group). * *p* < 0.05 and *** *p* < 0.001.

Next, we were interested in the impact of NRPs on the biological function of PAAD. Previous studies have studied the function of NRPs on immune cells, and its expression and activation on immune cells can inhibit anti-tumor immune response [9,22]. As described in Figure 2, the NRP proteins are also highly expression in PAAD tumor cells, so we investigated the effects of NRP proteins on the proliferation, invasion and migration of PAAD tumor cells. We constructed the NRP1 knockdown PANC-1 cell line and NRP2 knockdown CFPAC-1 cell line in vitro (Supplementary Figure S6A–D). Compared with normal PAAD cells, the proliferative (Figure 6A,B), invasive (Figure 6C,D), and migratory (Figure 6E,F) capacity of NRP-knockdown PAAD cells was impaired. Furthermore, the effects of the NRP1 inhibitor on the in vivo growth and progression of PAAD were investigated using the orthotopic pancreatic tumor-bearing nude (immunodeficient) and C57BL/6 (immunocompetent) mouse models (Figure 7). The results revealed that the NRP1 inhibitor suppressed tumor growth (*p* < 0.001, Figure 7B) and prolonged survival (*p* = 0.01, Figure 7D) compared with those in the control group of immunocompetent C57BL/6 mice. In immunodeficient nude mice, although no significant difference was observed in the survival time between the NRP1 inhibitor group and control group (*p* = 0.059, Figure 7C), some degree of improvement was observed, and the NRP1 inhibitor group showed some degree of reduction compared with the control group (*p* < 0.05, Figure 7A). These findings of in vivo experiments suggest that NRP1 depletion exerts anti-tumor effects and improves survival mainly via immune-related pathways.

**Figure 8:**
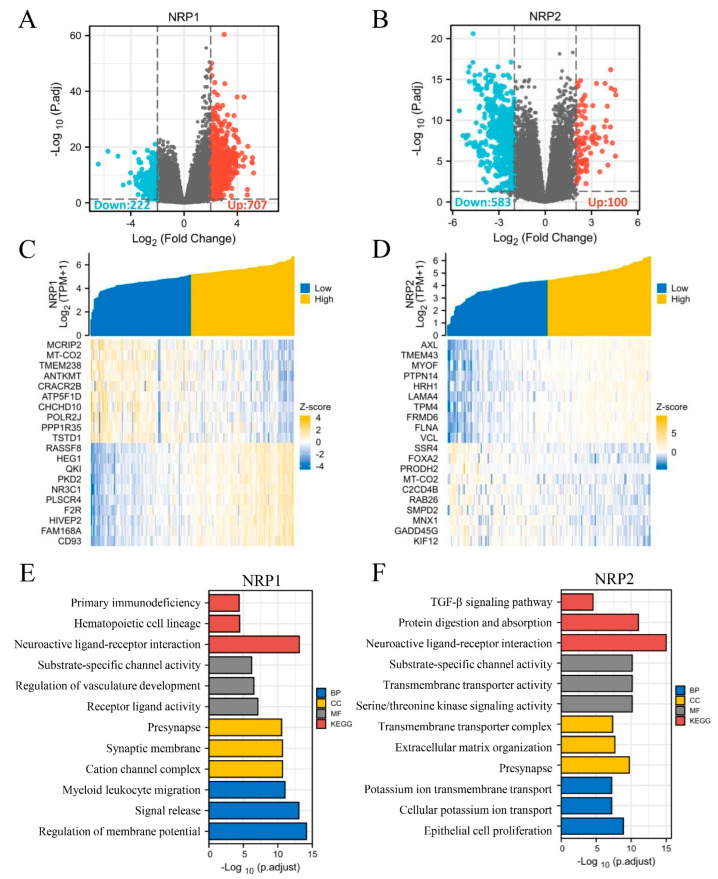
Differentially expressed genes of NRP and GO/KEGG analyses. (**A**,**B**) Volcano plot: red dots indicate significantly differentially up-regulated genes and blue dots indicate significantly differentially down-regulated genes. (**C**,**D**) Heatmap plot: the top 20 differentially expressed genes of NRPs. (**E**,**F**) GO/KEGG enrichment analysis. GO analysis contains biological pathways (BP), cellular components (CC) and molecular functions (MF).

In addition, based on the TCGA-PAAD dataset, we performed DEG analysis in silico using the DESeq2 R package. There were 929 and 683 DEGs for NRP1 and NRP2 expression, respectively, and the upregulated and downregulated genes are shown in volcano plots (|log_2_^(FC)^| > 2, adjusted *p* < 0.05) (Figure 8A,B). Given a large number of DEGs, only the top 20 upregulated and downregulated genes with the greatest differences have been presented in the heatmaps (Figure 8C,D). To explore the potential functions of NRP1- and NRP2-interactive DEGs, we performed functional enrichment analyses using GO/KEGG and GSEA. In addition to several known functions, such as nervous system development, angiogenesis, and lymphangiogenesis [5,6], some immune-related functions were also revealed. Specifically, NRP1 was significantly associated with primary immunodeficiency, myeloid leukocyte migration (Figure 8E), interleukin interactions, and inflammatory response regulation (Supplementary Figure S7). NRP2 was found to be associated with the TGF-β signaling pathway (Figure 8F). Interestingly, NRP1 was also involved in the PI3K-Akt signaling pathway and epithelial–mesenchymal transition (EMT) (Supplementary Figure S7). NRP2 was also involved in the Notch signaling pathway (Supplementary Figure S7) and transmembrane signal transduction (Figure 8F). Further in vivo studies are needed to verify whether NRPs can affect PAAD progression via these pathways, including, but not limited to, proliferation, invasion and migration mechanisms.

## 4. Discussion

In this study, we comprehensively investigated the roles of NRP1 and its homologous isoform, NRP2, using, but not limited to, bioinformatics methods for the first time. NRP expression, at both mRNA and protein levels, in tumor tissues was generally higher than that in normal tissues, which suggests that NRPs play a potential cancer-promoting role in most types of tumors. A subsequent study confirmed this conjecture: cancer patients with a high expression of NRPs had a worse OS and RFS, especially PAAD, which is in accordance with the results of Ben et al. [25]. In addition, our study showed the attractive diagnostic value of NRPs in almost all types of tumors. 

A key factor in the lethality of PAAD is acquired immune privilege, owing to an immunosuppressive TME and immune cell infiltration defects [26]. Based on the results of mIHC and single-cell RNA-seq of PAAD tumor tissue, the ubiquitous expression of NRPs in the TME was further revealed, and the results lay a foundation for the study of NRP depletion of targeted specific cells with anti-tumor effects. In fact, several studies have demonstrated that the infiltration and function of immune cells and regulation of immune checkpoints are partially mediated by NRP1 [10,11,21], as well as NRP2 [22,23]. Here, we employed bioinformatics methods with multiple databases to analyze the role of NRP tumor immunology in PAAD and pan-cancer. An important finding was a significant positive correlation between the expression of NRPs and infiltration level of innate immune cells in many cancers, including PAAD. Emerging studies have reported the antitumor effects of anti-NRP1 [27] and -NRP2 [22], mediated by tumor-associated macrophages. The roles of NRPs in the function of innate immune cells and the targeted therapy based on it may be worthy of further research. Some studies have also revealed the important role of B cells, CD4^+^ Th cells, and NKT cells in the proliferation and differentiation of cytotoxic T lymphocytes and the enhancement of antitumor immunity [28,29,30]. Interestingly, our study suggests that the expression of both NRP1 and NRP2 is negatively correlated with the infiltration of these cells in almost all cancers. These findings highlight the inhibitory role of NRPs in antitumor immunity in these cells. Treg cells can help tumor cells escape killing by cytotoxic CD8^+^ T cells, by exerting an immunosuppressive effect [31], and in the context of hepatocellular carcinoma, NRP1 supports Treg cells’ migration behavior, and anti-NRP1 showed a favorable and safe outcome and evoked the antitumor effect of PD-1 blockade [32]. Our results showed a positive correlation between Treg infiltration levels, and NRP1 and NRP2 expression was observed in PAAD, which partially revealed the immunosuppressive TME and poor prognosis of tumor patients with high expression of NRP1 and NRP2. Interestingly, the higher expression of NRP1 seems to imply more CD8^+^ T cell infiltration. A possible explanation is that although the number of CD8^+^ T cells is large, their function may be limited. PAAD is characterized by dense stromal deposition, which is mainly caused by infiltrated CAF [33]. Our results also showed that the expression of NRP1 and NRP2 was positively correlated with the infiltration of CAF, which can interact with tumor cells, promote tumor growth and maintain malignant tendency [33].

Independent of the number of infiltrated T cells, one of the main causes of immunosuppression in the TME is T-cell exhaustion, which is defined by effector function defects and sustained expression of inhibitory receptors [34]. Here, we confirmed that the expression of NRPs, especially NRP1, was positively correlated with most of the immune checkpoint expression in nearly all cancers, including PAAD. As immune checkpoint candidates, NRPs may also be involved in T-cell exhaustion and tumor immune evasion by orchestrating multiple immune checkpoints. In recent years, traditional ICB therapies, such as anti-PD-1/-L1 and -CTLA4, have shown improved antitumor efficacy in some patients with cancer [3,4]. The pessimistic response in certain cancers, such as PAAD, limits its application. Our study demonstrated that a high expression of NRP1 means a low ICB response in PAAD, using TIDE assessment. Together, these findings promote the development of NRP-based prediction methods for ICB response and combination with ICB therapies.

In the GO/KEGG analyses, GSEA and in vitro experiments, we partially proved the mechanisms by which NRPs exert protumor effects via immune-related and -independent interactions. NRP1 is involved in primary immunodeficiency, hematopoietic cell lineage regulation, and myeloid leukocyte migration, which may explain the positive correlation between NRP1 expression and myeloid immune cell infiltration. In other words, NRP1 may promote the generation (differentiation and maturation) and migration of myeloid immune cells to the TME, thereby impairing antitumor immunity. However, whether these myeloid immune cells play a function similar to myeloid-derived suppressor cells remains to be studied. NRP2 is associated with TGF-β signaling, unlike NRP1, which is associated with interleukins and complement regulation, which outlines the complex cytokine milieu in the TME mediated by NRPs. It is also necessary to study the interaction between NRPs and cytokines within the TME in the future.

Regarding immune-independent interactions, our study indicated that NRP1 and NRP2 silencing impairs the proliferation, invasion, and migration of human PAAD cells in vitro. In addition, NRP1 is involved in the PI3K-Akt and EMT signaling pathways, which is highly reminiscent of previous research showing that NRP1 can induce EMT to enhance the migration and invasion ability of gastric cancer cells by activating the PI3K/Akt signaling pathway [35]. Moreover, the latest literature also indicated that NRP1 promotes prostate cancer progression via modulating the EGFR-dependent AKT pathway activation [36]. Further in vivo experiments revealed that the NRP1 inhibitor suppressed PAAD tumor growth and prolonged survival, and the effects were more pronounced in the immunocompetent C57BL/6 mouse model. The results highlighted that NRP1 depletion exerts antitumor effects mainly via immune-related pathways. It is an important and promising finding, and the results of bioinformatics analysis may partially reveal its mechanism, necessitating further research.

## 5. Conclusions

With this comprehensive understanding, there are strong reasons to believe that NRPs, especially NRP1, are attractive diagnostic and prognostic biomarkers that mediate immunoregulation and have functions beyond immunology in the context of cancer. As potential novel immune checkpoints, NRPs provide a new opportunity for tumor immunotherapy, and they will be of interest for further study. In the future, to overcome the limitations of our study, more preclinical and clinical studies are needed to prove the multiple antitumor roles of NRPs.

## Data Availability

The datasets generated and analyzed during the current study are available from the corresponding author on reasonable request.

## Supplementary Materials

The following supporting information can be downloaded at: https://www.mdpi.com/article/10.3390/cancers15082225/s1, Supplemental Materials and Methods: Western blot (WB), immunohistochemistry (IHC), multiplexed immunohistochemistry (mIHC), animal study, cell culture and construction of NRP-knockdown cells, quantitative real-time polymerase chain reaction (qRT-PCR), cell proliferation, invasion, and migration assays. Figure S1A: NRP mRNA expression between tumor tissues from the TCGA database and normal tissues from the TCGA and GTEx databases in pan-cancer; Figure S1B: The expression of NRP1 and NRP2 proteins from the HPA database. Figure S2A,B: NRP-related “protein–protein interaction” network in PAAD tissue from the GENEMANIA database; Figure S2C: ROC curve analysis evaluating the performance of NRPs for a string of cancer diagnoses. Figure S3: The mRNA expression of NRPs in PAAD tumor microenvironment. Figure S4: Immune cell infiltration analysis in PAAD; Figure S5: The line charts represent the correlation between NRP expression and immune check point expression in PAAD; Figure S6: Validation of NRP knock-out CFPAC-1 cell lines. Figure S7: GSEA analysis of NRPs in PAAD. Figure S8: All original images.

## Abbreviations

ICB: immune checkpoint blockade; NRPs: Neuropilins; PAAD: pancreatic adenocarcinoma; TCGA: The Cancer Genome Atlas; GTE: Genotype-Tissue Expression; GO: Gene Ontology; KEGG: Kyoto Encyclopedia of Genes and Genomes; GSEA: gene set enrichment analysis; TME: tumor microenvironment; IHC: immunohistochemistry; mIHC: multiplexed immunohistochemistry.
