# Supplementary material for: Immune Checkpoint Neuropilins as Novel Biomarkers and Therapeutic Targets for Pancreatic Cancer
Source: Cancers (Basel). 2023 Apr 10;15(8):2225. doi: 10.3390/cancers15082225 (PMC10136637; doi:10.3390/cancers15082225)
Supplement: Supplementary file 1 [file cancers-15-02225-s001.zip › cancers-2289784-supplementary.pdf]

cancers

MDPI

Supplementary Materials

Immune Checkpoint Neuropilins as Novel Biomarkers and Therapeutic Targets for Pancreatic Cancer

Li-Hong He, Xiao-Zhen Zhang, Meng-Yi Lao, Han-Jia Zhang, Han-Shen Yang, Xue-Li Bai

S1. Supplemental Materials and Methods

S1.1. Western blot (WB)

For WB, radioimmunoprecipitation assay (RIPA) buffer (P0013B Beyotime Biotechnology, Shanghai, China) containing phenylmethanesulfonylfluoride (ST505 Beyotime Biotechnology) was used to lyse cells for 30 min on ice, followed by centrifugation at 12000g for 15 min at 4°C. The supernatant was collected containing the soluble proteins. The protein concentration was measured using the bicinchoninic acid (BCA) reagent (P0012 Beyotime Biotechnology). The lysates were heated at 100 °C in NuPAGE LDS Sample Buffer (4x) (Thermo Fisher Scientific, Waltham, MA, USA) for 3–5 min, separated using sodium dodecylsulfate polyacrylamide gel electrophoresis before being transferred electrophoretically to a polyvinylidene fluoride membrane (Millipore, Billerica, MA, USA). 5% skim milk in TBST was used to block the membranes, which were the overnight at 4 °C with primary antibodies. After washing with TBST, the membrane was incubated at 4 °C for 2 h with species-specific secondary antibodies. The signals from the immunoreactive proteins were detected using an EzWay DAB Western Blot Kit (KOMA BIOTECH, Seoul, Korea) and the correspondent bands were visualized using ChemiScopeTouch (Clinx Science Instruments, Shanghai, China). The internal controls comprised α-tubulin (1:2000 dilution, AF5012, Beyotime Biotechnology, Shanghai, China). NRP1 (1:1000 dilution, EPR3113) and NRP2 (1:1000 dilution, EPR23808-72) antibodies were purchased from Abcam (Cambridge, MA, USA). The intensity of immunoblotting bands was measured using Image J 1.8.0(National Institutes of Health, Bethesda, MD, USA).

S1.2. Immunohistochemistry (IHC)

Paraffin-embedded PDAC samples were obtained from the Department of Hepatobiliary and Pancreatic Surgery, the First Affiliated Hospital, Zhejiang University School of Medicine. Four μm-thick sections were cut and placed onto glass slides, baked for 60–90 min at 68 °C, and then deparaffinized. The antigen was retrieved using Sodium Citrate Antigen Retrieval Solution (Solarbio Life Science, Beijing, China) and the sections were boiled for 10 min, followed by incubation for 25-30 min at room temperature. 3% BSA was used to block the samples for 30 min at room temperature. Each tissue sample was treated with primary antibodies, as indicated, and incubated overnight at 4 °C, followed by incubation with a biotin-conjugated secondary antibody for 50 min at room temperature. Primary antibodies comprised NRP1 (EPR3113, 1:300 dilution, Abcam) and NRP2 (sc-13117, 1:50 dilution, Santa Cruz). The target protein was visualized using a diaminobenzidine (DAB) Chromogen Kit (BDB2004; Biocare Medical; Pacheco, CA, USA), where the brown color characterized the targeted molecule. Slides were counterstained with diluted hematoxylin for 3–5 min. Representative images per samples were captured using Image Scope software (Leica Biosystems, Wetzlar, Germany).

S1.3. Multiplexed immunohistochemistry (mIHC)

The mIHC was performed by staining 4-um-thick formalin-fixed, paraffin-embedded whole tissue sections with standard, primary antibodies sequentially and paired with TSA 7-color kit (D110071-50T, Yuanxibio). Then by staining with DAPI. For example,

deparaffinized slides were incubated with anti-panCK (AE1/AE3) antibody (#GM351507, Gene tech), for 60 minutes and then treated with Alexa Fluor 488 goat anti-mouse IgG(H+L) peroxidase-conjugated (HRP) secondary antibody (#A10011-60, Yuanxibio) for 60 minutes. Then add DAPI for nuclear stain for 15mintes, Scanning under a fluorescence microscope. Complete elution after photographing. Start the second to sixth round of staining，Slides were washed in TBST buffer and then transferred to preheated EDTA solution (100°C) before being heat-treated using a microwave set at 20% of maximum power for 15 minutes. Slides were cooled in the same solution to room temperature. incubated with anti-CD8 (#BX50036-C3) for 60 minutes and then treated with peroxidase-conjugated (HRP) secondary antibody (#DS9800, Leica) for 10 minutes. Then labelling was developed for a strictly observed 10 minutes, using TSA 620 per manufacturer's direction. Between all steps, the slides were washed with Tris-buffer. The same process was repeated for the following antibodies/fluorescent dyes, in order: anti-CD68 (#BX50031, Biolynx)/ TSA 520， anti-a-SMA (#19245s, CST)/ TSA 670, anti-CD4 (#ab133616, abcam)/ TSA 570, anti-NRP1 (#ab81321, abcam)/ TSA 440 and anti-NRP2 (#HPA039980, Atlas)/ TSA 440. Each slide was then treated with 2 drops of DAPI (D1306; Thermofisher), washed in distilled water, and manually coverslipped. Slides were air dried, and take pictures with Pannoramic MIDI tissue imaging system (3DHISTECH). Images was analyzed using Indica Halo software.

S1.4. Animal Study

Male 6–8-week-old C57BL/6 mice and nude mice were purchased from the Model Animal Research Center of Nanjing University and housed in specific pathogen-free conditions in cages of up to five animals. All mice were maintained under a 12-hour dark/12-hour light cycle with food and water provided ad libitum. All animal experiments were reviewed and approved by the Ethics Committee of the First Affiliated Hospital, School of Medicine, Zhejiang University.

S1.5. Cells culture and construction of NRPs-knockdown cells

KPC cells from the genetically engineered mouse model (GEMM-KPC) (LSL-KrasG12D/+; LSL-Trp53R172H/+; Pdx1-Cre) mice were used in the animal experiments, which were kindly donated by the laboratory of Prof. Raghu Kalluri (MD Anderson Cancer Center, Houston, TX, USA). KPC cells were grown in modified McCoy's 5A Medium (Thermo Fisher Scientific, Waltham, MA, USA) with 10% fetal bovine serum (Gibco, USA) and 1% penicillin-streptomycin (100 IU/mL; Gibco,USA) with 5% CO2 at 37°C.

Human pancreatic cancer cells CFPAC-1 and PANC-1 were a gift from the Liang Laboratory and cultured under standard condition containing DMEM medium (for PANC-1) or PMRI 1640 medium (for CFPAC-1) (Gibco, Carlsbad, USA) supplemented with 1% Penicillin-Streptomycin (100 IU/mL; Gibco, USA) and 10% fetal bovine serum (Gibco, USA) with 5% CO2 at 37°C.

The shNRP1 and shNRP2 plasmids were purchased from OBiO (shanghai, China): sh1NRP1:5′-AAAGCCCCGGGTACCTTACAT-3′; sh2NRP1:5′-CAGCCTTGAATGCACTTATAT-3′; sh3NRP1:5′-TATACTAGAATCACCGCATTT-3′; sh1NRP2:5′-CCTCAACTTCAACCCTCACTT-3′; sh2NRP2:5′-CGTTTCCAGATGACAGGAATT-3′; sh3NRP2:5′-CGACTGCAAGTATGACTTTAT-3′. According to the manufacturer's instructions, shNRP1 plasmids were transfected into PANC-1 cells and shNRP2 plasmids were transfected into CFPAC-1 cells, with JetPRIME Polyplus (Illkirch Graffenstaden, France). The efficiency of transfection was determined using WB and qRT-PCR to evaluate protein and mRNA expression after cell collection.

S1.6. Quantitative real-time polymerase chain reaction (qRT-PCR)

Total RNA from the cell lines were extracted using TRIzol reagent (TaKaRa, Dalian, China), according to the manufacturer's instructions. RNA concentration was detected by Nano Drop 1000 (Thermo Fisher Scientific, USA), and complementary DNA was synthesized with a Prime Script RT reagent kit (Takara, Japan) using 2 μg RNA. SYBR-Green dye

(TaKaRa) and Corbett Rotor-Gene 3000 thermocycler were used to perform the qRT-PCR reaction, according to the manufacturer's protocol. The amplification conditions of quantitative qRT-PCR were set as follows: 95 ˚C for 30 s, 95 ˚C for 5 s, 60 ˚C for 34 s, 95 ˚C for 15 s and a total of 40 cycles. The experiments were repeated in triplicate. Analysis of the Cycle threshold (Ct) value suggested the difference between mRNA expression levels of NRPs genes in each group. NRP1, NRP2 and GAPDH primers were synthesized and purchased from SunYa (Hangzhou, China). The sequences of the primers were: for NRP1: sence, 5′-ATCACGTGCAGCTCAAGTGG-3′ and antisence, 5′-TCATGCAGTGGG-CAGAGTTC-3′; for NRP2: sence: 5′-GCTGGCTATATCACCTCTCCC-3′ and antisence, 5′-TCTCGATTTCAAAGTGAGGGTTG-3′; for GAPDH: sence, 5′-GGTATGACAAC-GAATTTGGC-3′ and antisence, 5′-GAGCACAGGGTACTTTATTG-3′.

S1.7. Cell proliferation, invasion, and migration assays

For the CCK-8 assay, about one thousand viable cells per hole were placed into 96-well plates in a final volume of 100 μl/well. Every 24 h, 10 μl of CCK-8 solution was added to each well, and the plate was further incubated for 2 h at 37 ˚C. Absorbance at 450 nm was quantitated using a microplate reader. The experiment was performed for 5-7 days, and a cell growth curve was drawn with the experimental data. PAAD cells invasive capacity was assessed by transwell assay using Corning BioCoat Matrigel Invasion Chambers with BD Matrigel Matrix (Thermo Fisher Scientific) according to the manufacturer's instructions. First, cells (1×10^4/chamber) with 200 μl serum-free medium were added to the upper compartment, and 600 μl DMEM or RPMI 1640 medium containing 20 % FBS was added to the lower compartment, and incubated for 36-72 h at 37 °C. After incubation, the cells in the upper chamber migrated to the lower surface of the membrane. The cells on the upper membrane were removed carefully with a cotton tip; the penetrated polycarbonic membrane was fixed with 4 % neutral poly formaldehyde for 30 min, and stained with 0.1% crystal violet for 30 min. The number of migrated cells was counted in 6 randomly selected fields under an inverted microscope. Independent experiments were performed in triplicate. For the wound healing assay, tumor cells were seeded in 6-well plates at a density and incubated till 70–80 % confluence as a monolayer. A cell-free straight line was scratched at the center of the well with a sterile 1000-μl pipette tip. Another straight line was scratched perpendicular to the first line to produce a cross-shaped cellular gap in each well. The cells were subsequently washed twice with PBS and refreshed with serum-free medium. The scratch was photographed under a fluorescence microscope every 12 h until it was filled with cells. Digital images of the cell gap were captured at different time points under the microscope.

S2. Supplemental Figures

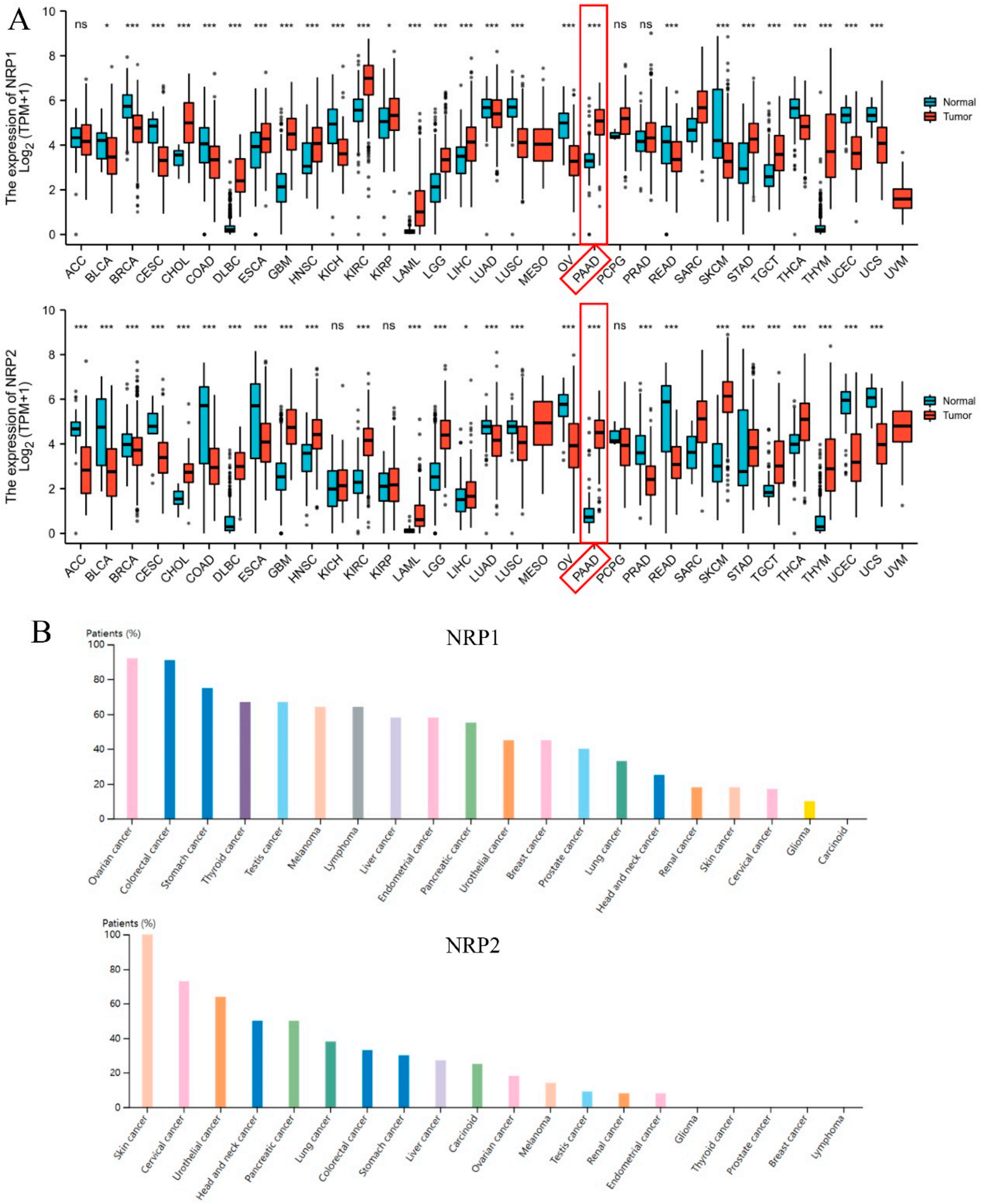

Figure S1. (A) NRPs mRNA expression between tumor tissues from TCGA database and normal tissues from TCGA and GTEx database in pan-cancer. **(B)** The expression of NRP1 and NRP2 protein from HPA database.

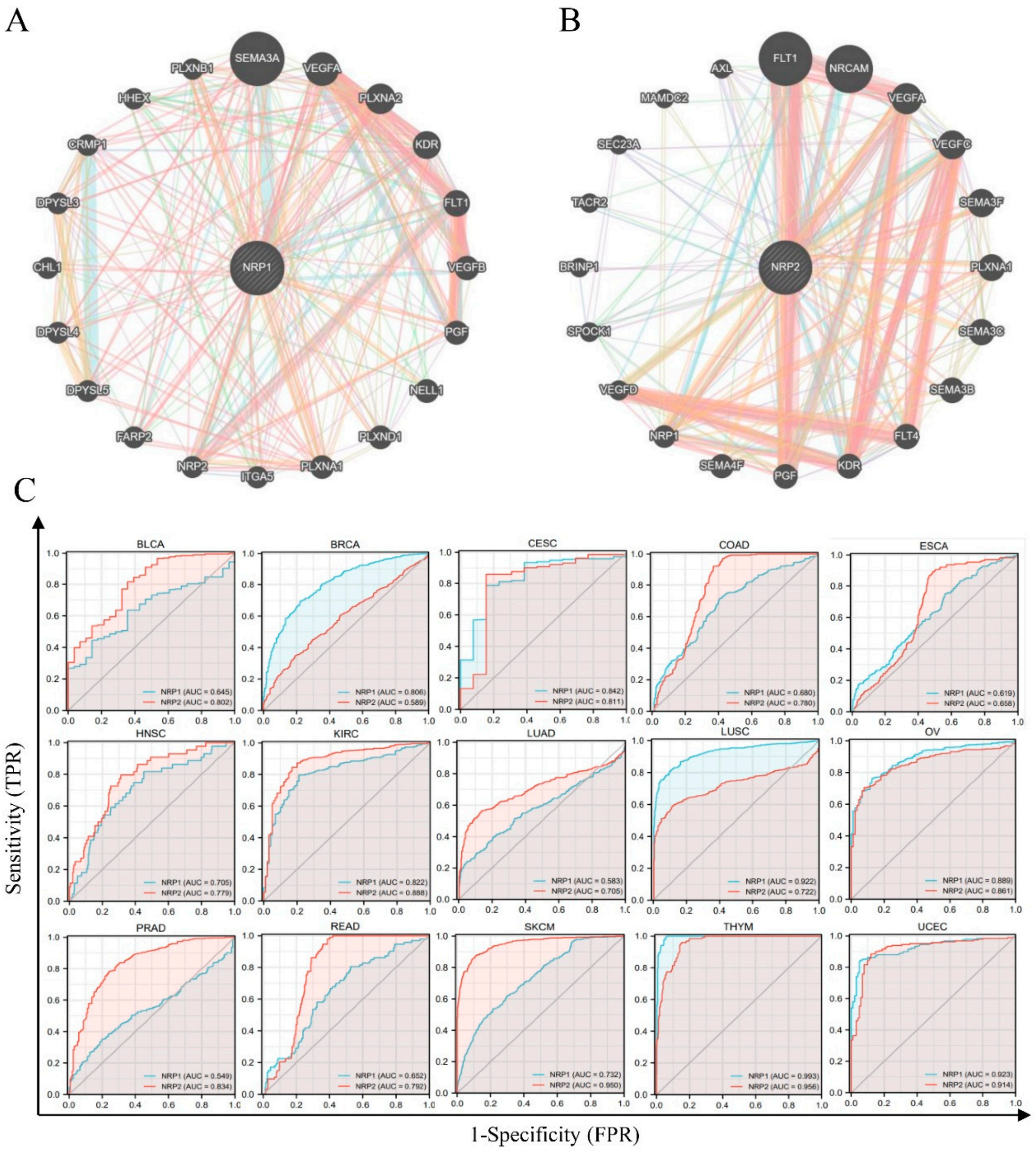

Figure S2. (A-B) NRPs-related "protein-protein interaction" network in PAAD tissue from GENEMANIA database. **(C)** ROC curve analysis evaluating the performance of NRPs for a string of cancers diagnosis.

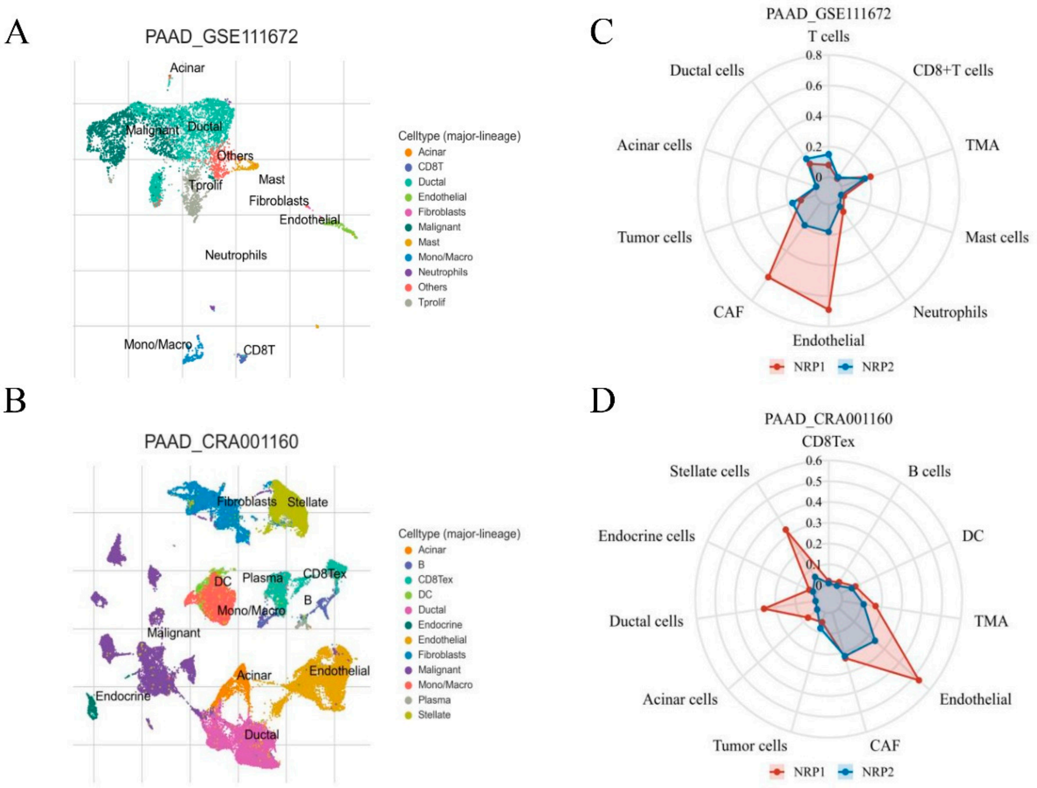

Figure S3. The mRNA expression of NRPs in PAAD tumor microenvironment. **(A-B)** The detailed cell-type annotation at the single-cell level of two PAAD datasets from TISCH database. **(C-D)** The radar charts show the expression of NRPs in different cell types across the two datasets.

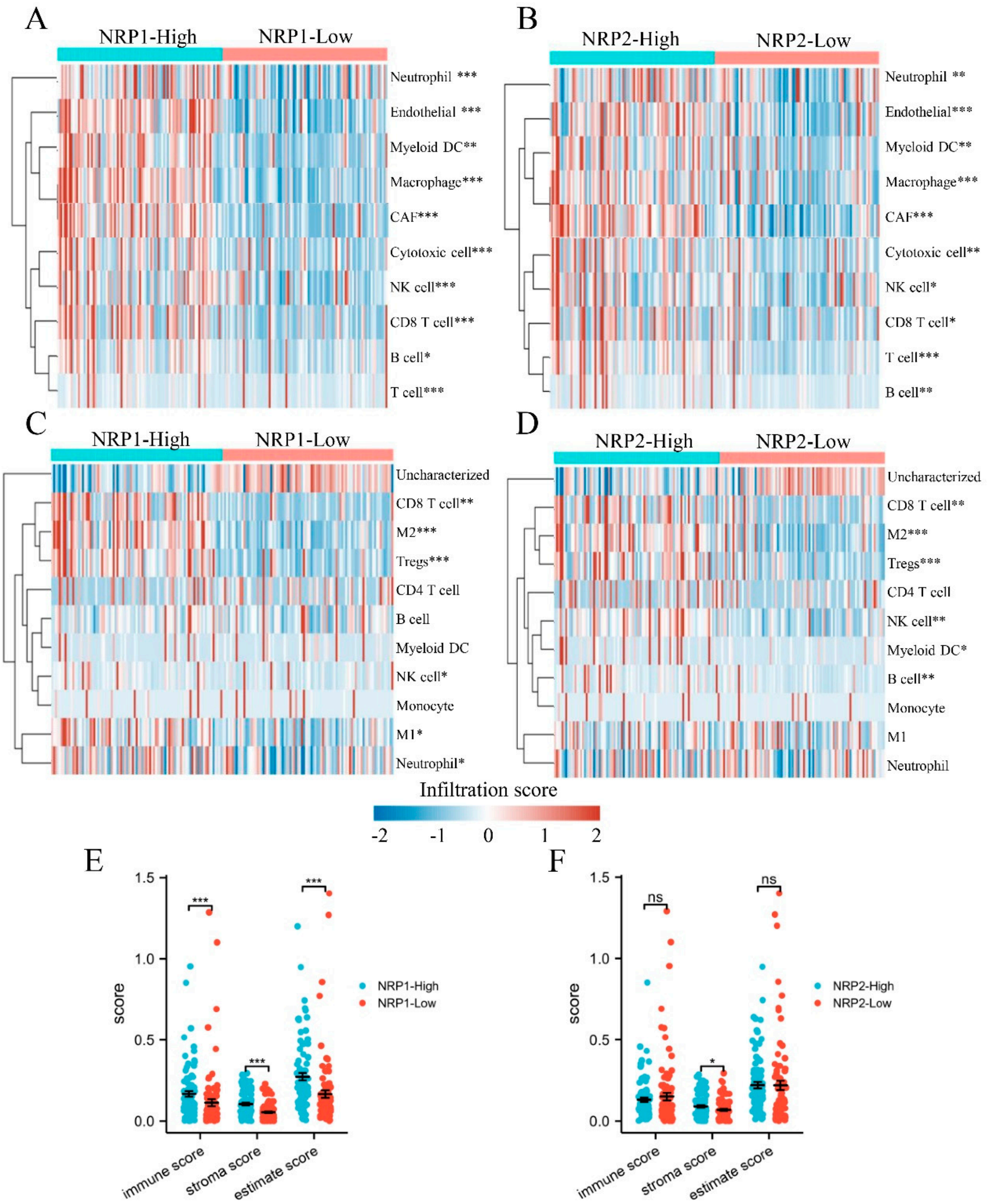

Figure S4. Immune cell infiltration analysis in PAAD. **(A, B)** The correlation between NRP1 as well as NRP2 and the immune cells infiltration level using MCP-counter. **(C, D)** The correlation between NRP1 as well as NRP2 and the immune cells infiltration level using QuantTIseq. **(E-F)** The total abundance of immune and stromal cells of individual PAAD sample by ESTIMATE method. *p < 0.05; **p < 0.01 and ***p < 0.001; ns, not significant.

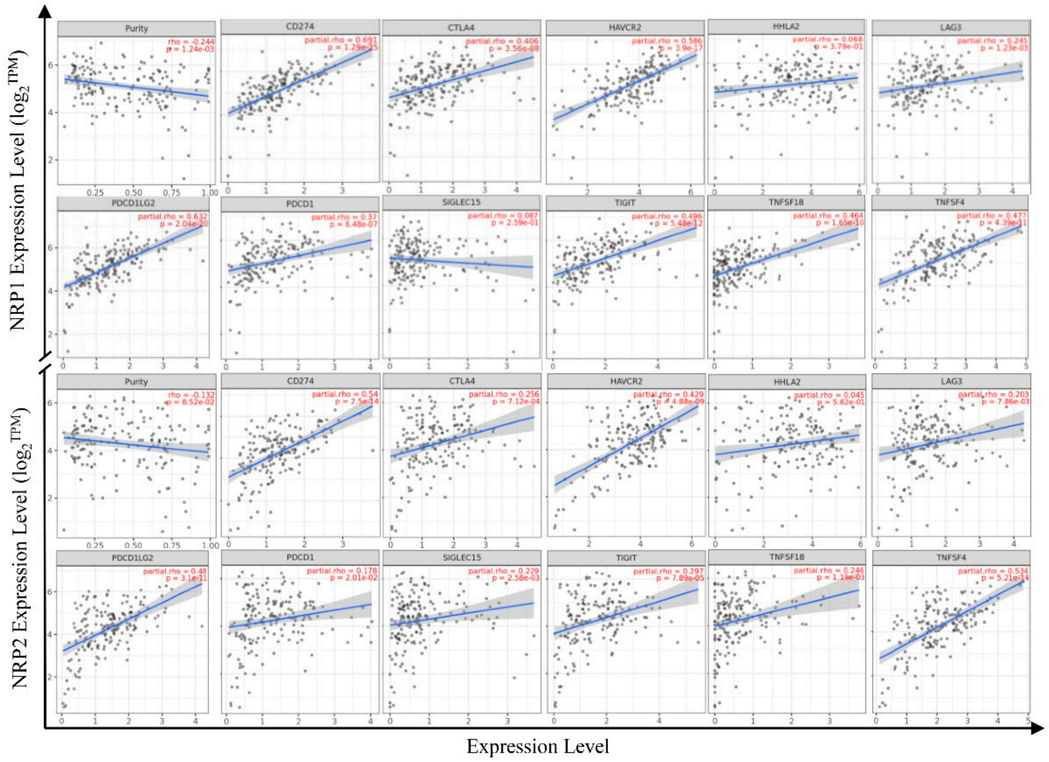

Figure S5. The line charts represent the correlation between NRPs expression and immune check points expression in PAAD.

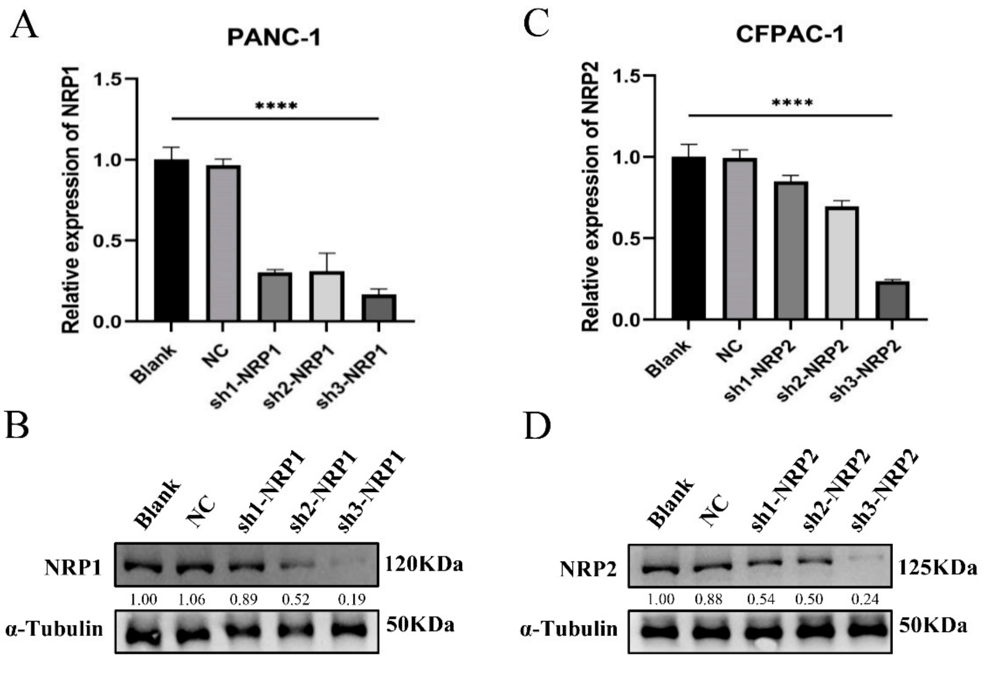

Figure S6. Validation of NRPs knock-out CFPAC-1 cell lines. Expression of NRP1 **(A)** and NRP2 **(C)** mRNA in PANC-1 and CFPAC-1 cells, respectively, after transfection, as detected with qRT-PCR. Expression of NRP1 **(B)** and NRP2 **(D)** protein in PANC-1 and CFPAC-1 cells, respectively, after transfection, as detected with Western blot. NC, negative control; Blank, not transfected. ****p < 0.0001.

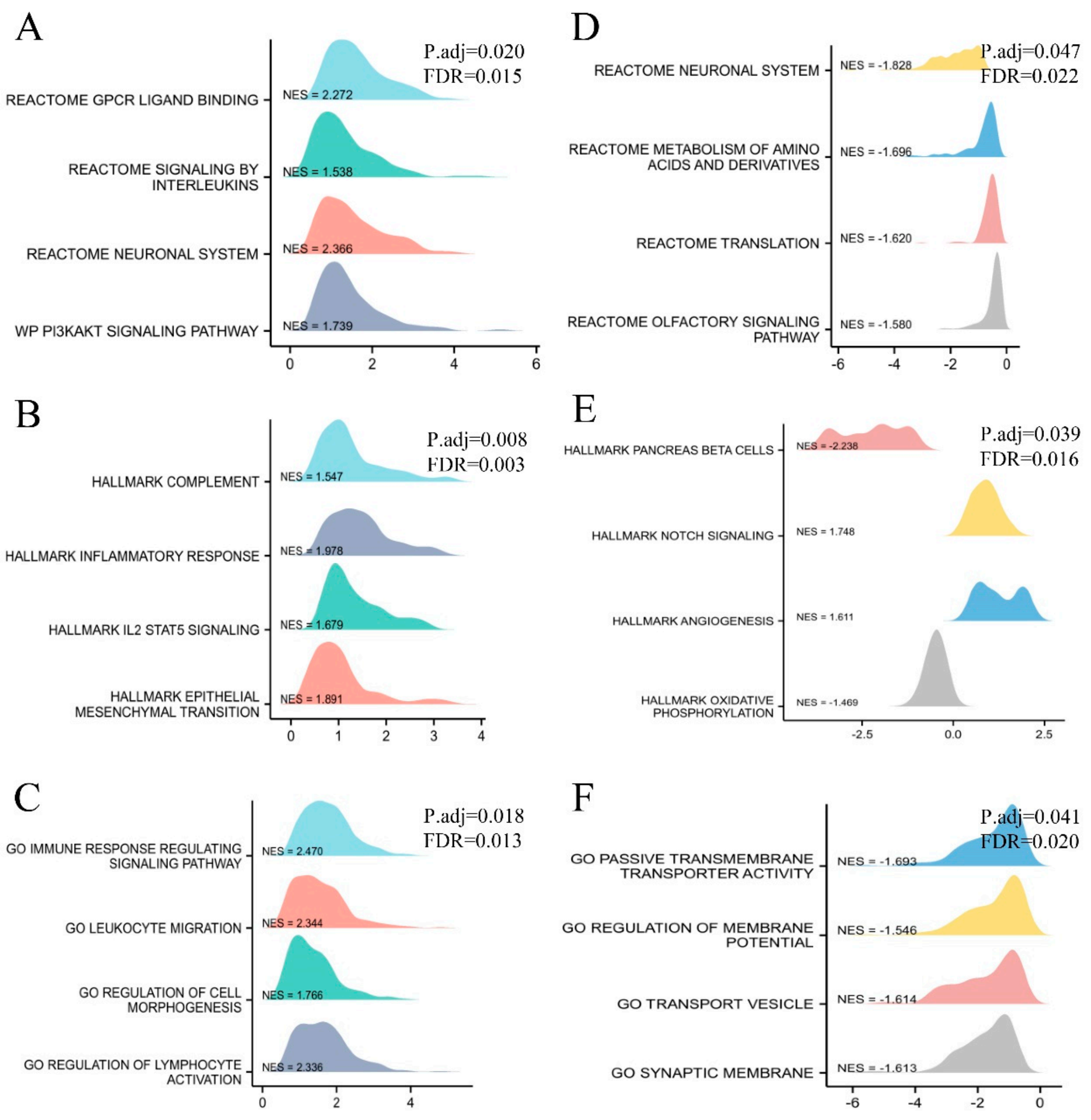

Figure S7. GSEA analysis of NRPs in PAAD. **(A-C)** GSEA enrichment analysis of NRP1. **(D-F)** GSEA enrichment analysis of NRP2.

NRP1

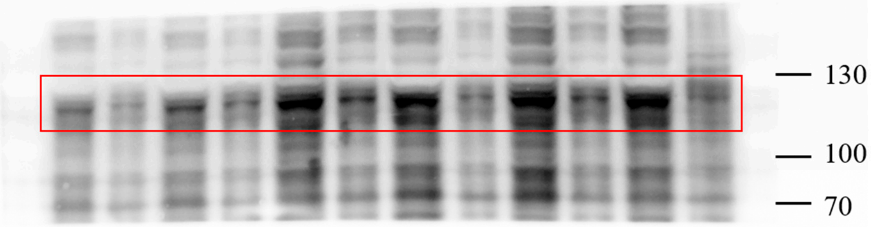

α-Tubulin

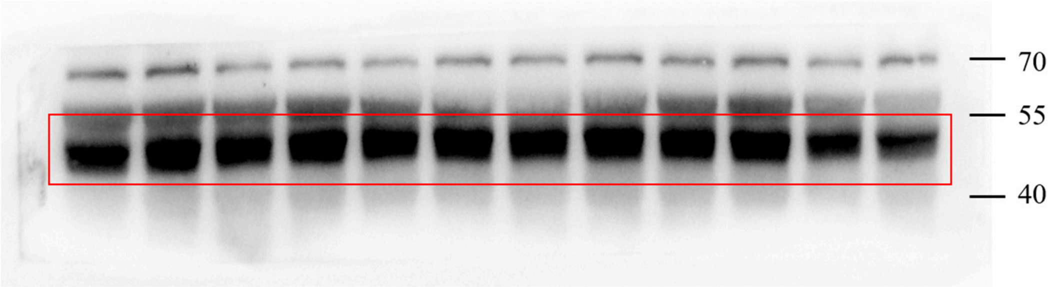

NRP2

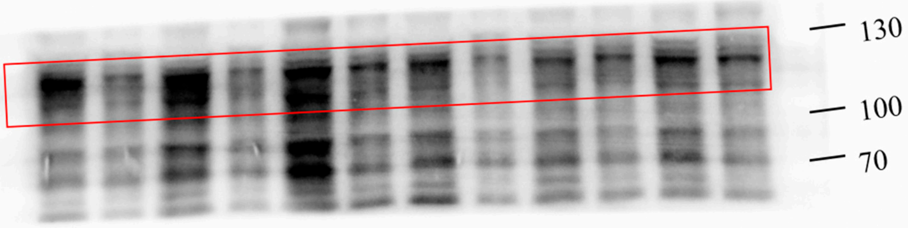

α-Tubulin

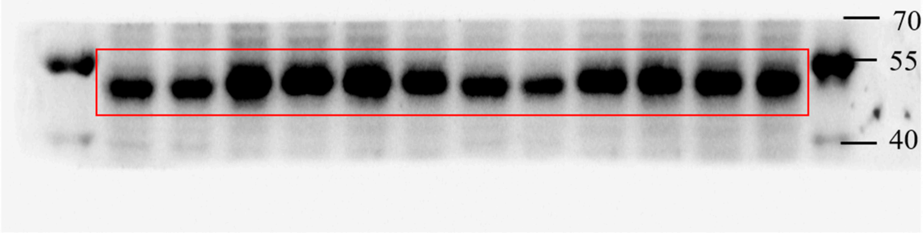

NRP1-normal

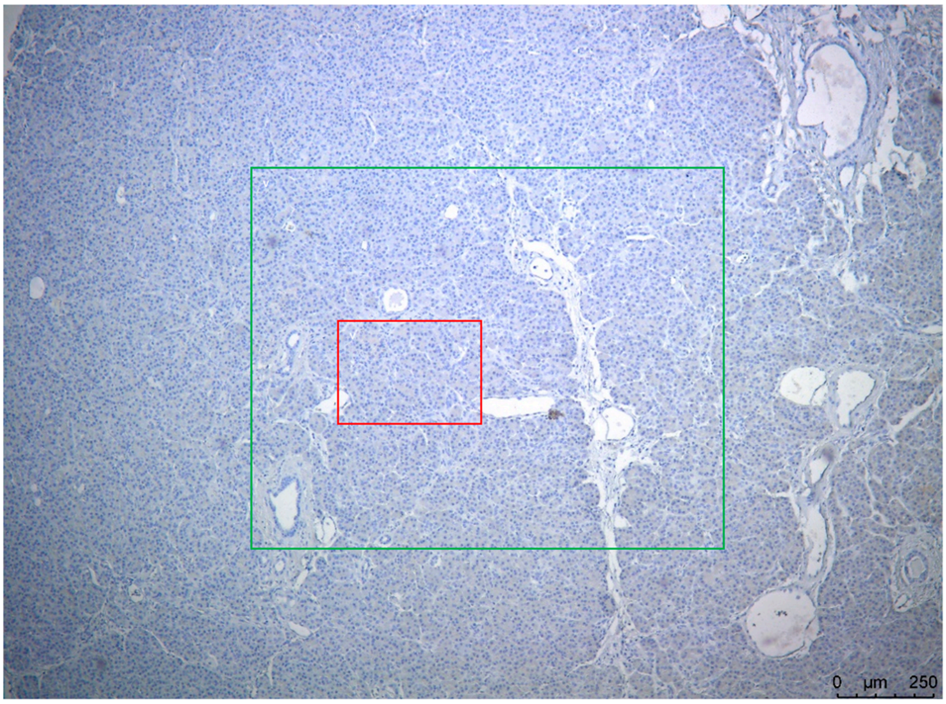

NRP1-tumor

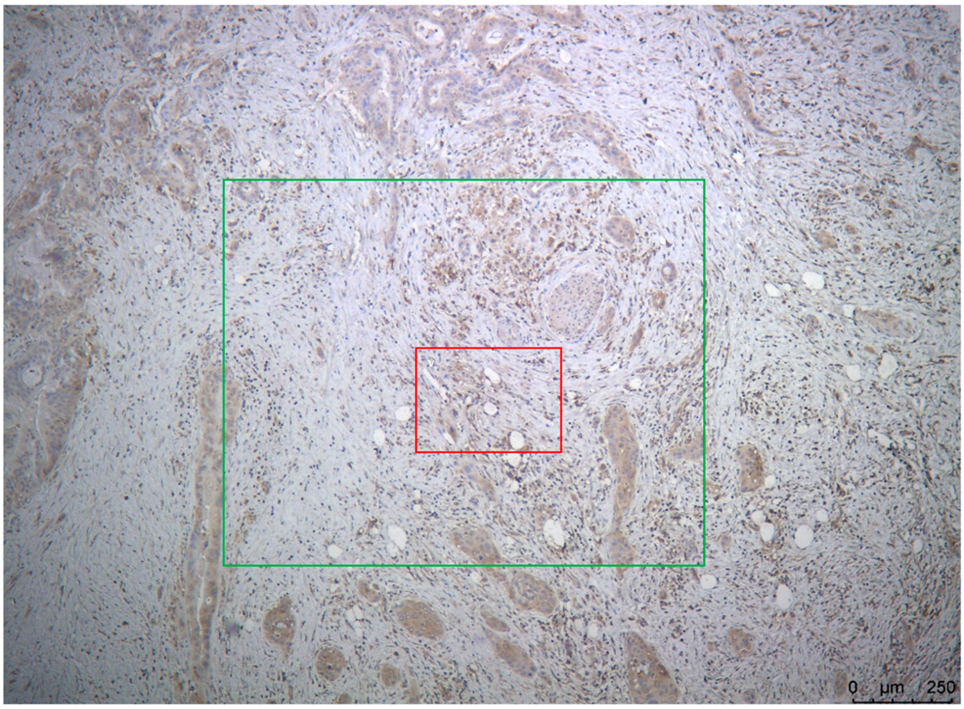

NRP2-normal

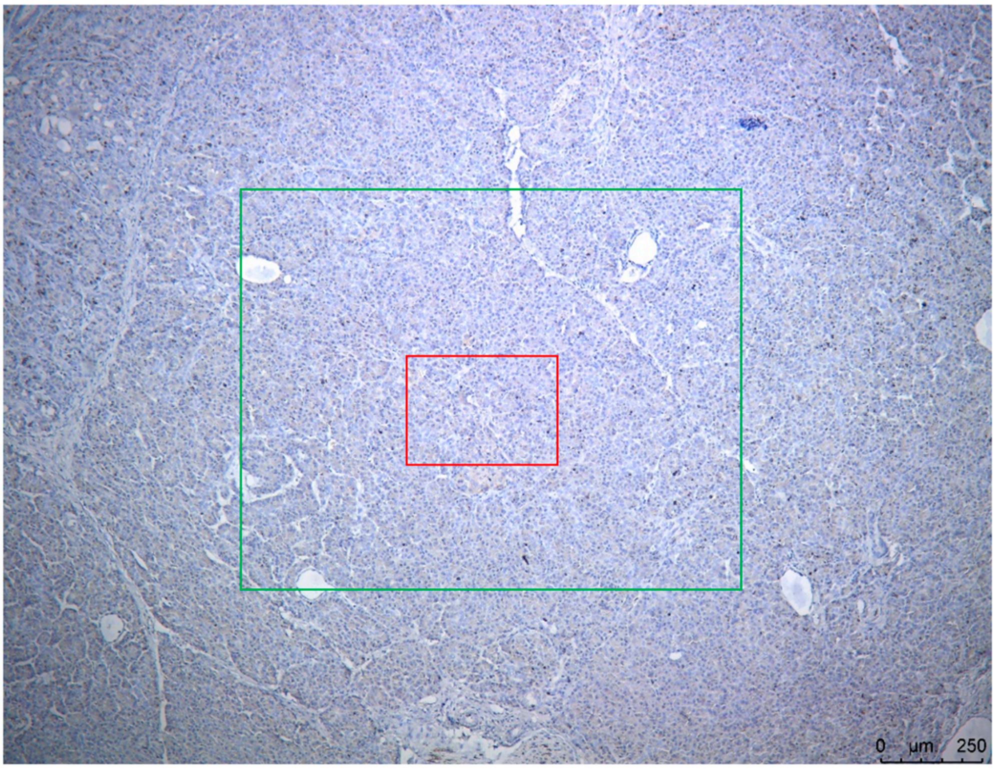

NRP2-tumor

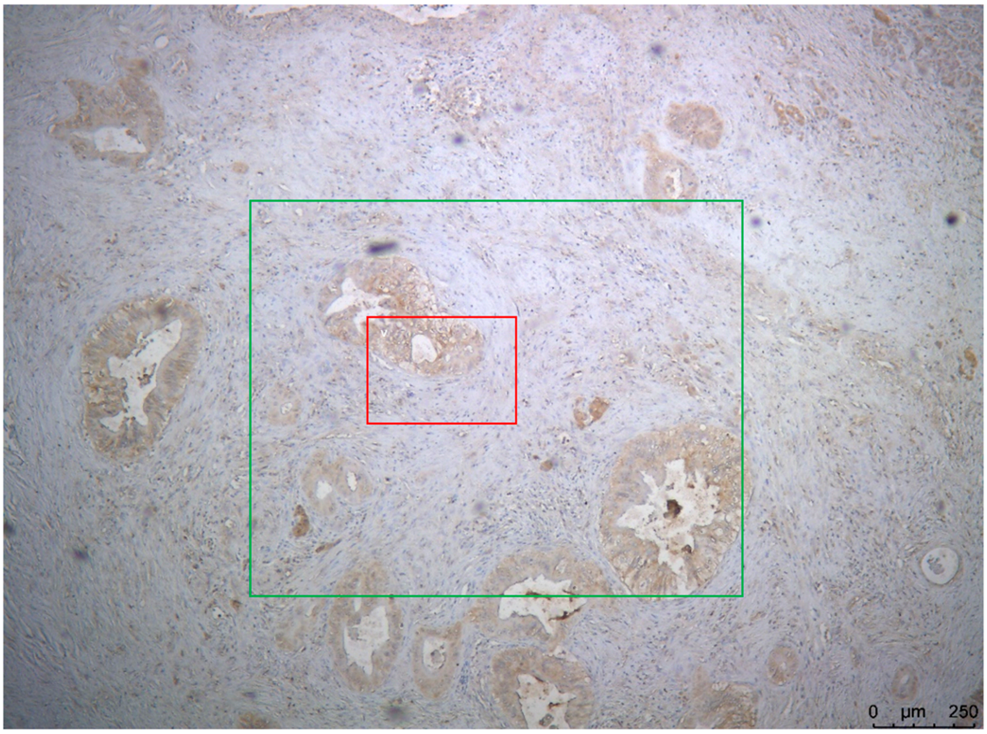

NRP1-mIHC

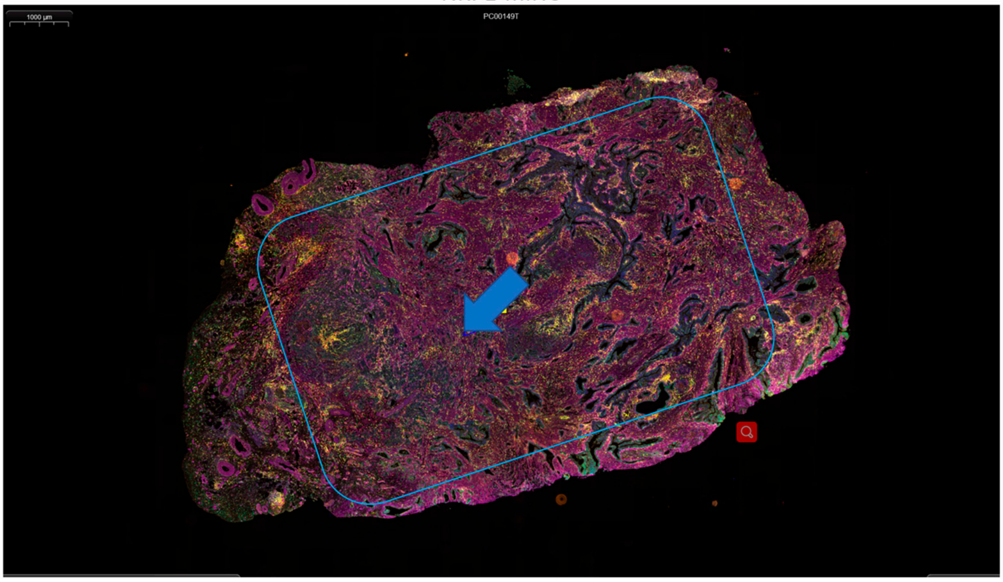

The original image exceeds 500MB, which is provided as a link, https://www.jianguoyun.com/c/sd/1580423/b2e5cb9d03eeb32, and downloaded to CaseViewer for viewing.

NRP2-mIHC

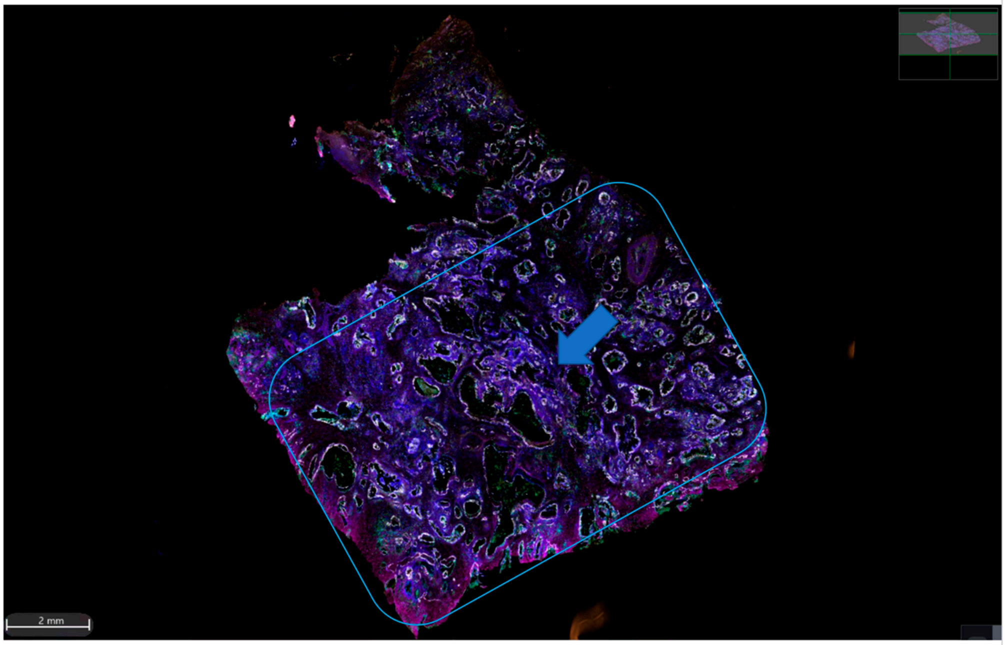

The original image exceeds 500MB, which is provided as a link, https://www.jianguoyun.com/c/sd/1580423/b2e5cb9d03eeb32, and downloaded to CaseViewer for viewing.

PANC1: NRP1-NC

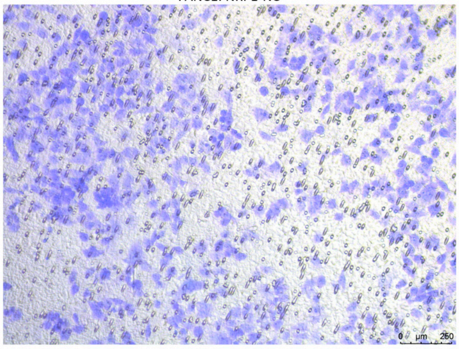

CFPAC1: NRP2-NC

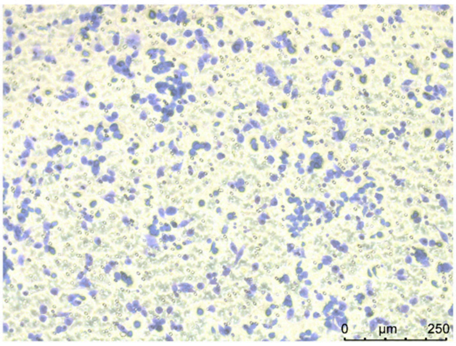

PANC1: NRP1-BLANK

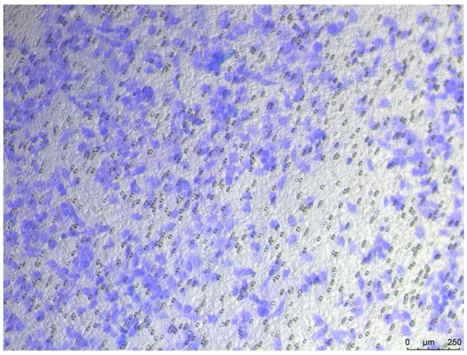

CFPAC1: NRP2-BLANK

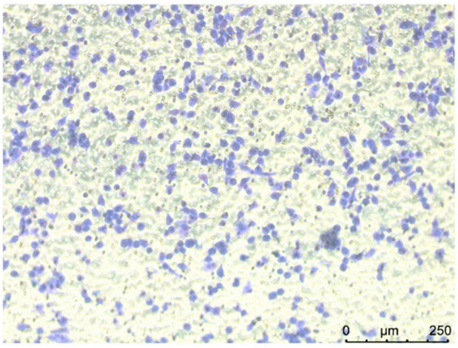

PANC1: NRP1-sh3

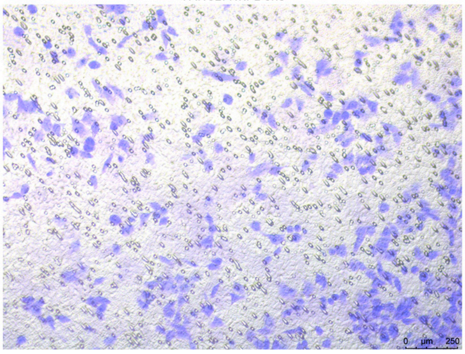

CFPAC1: NRP2-sh3

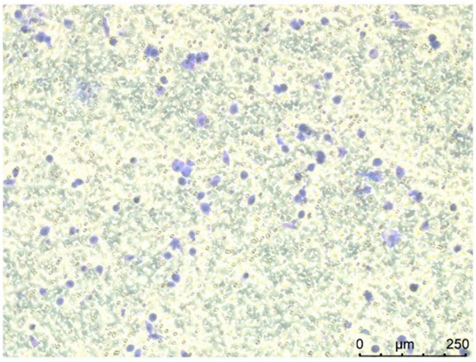

FigS6B

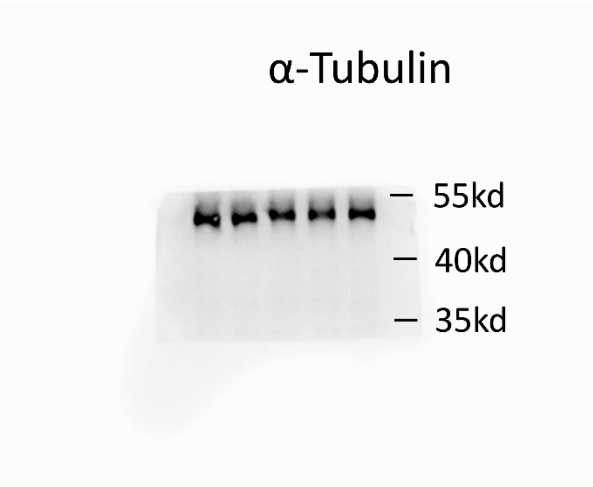

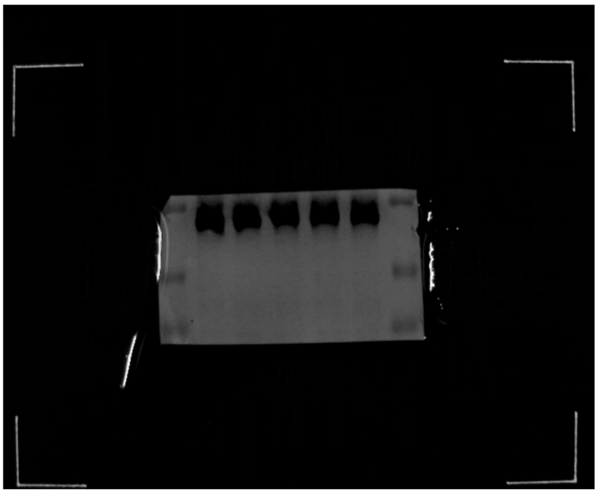

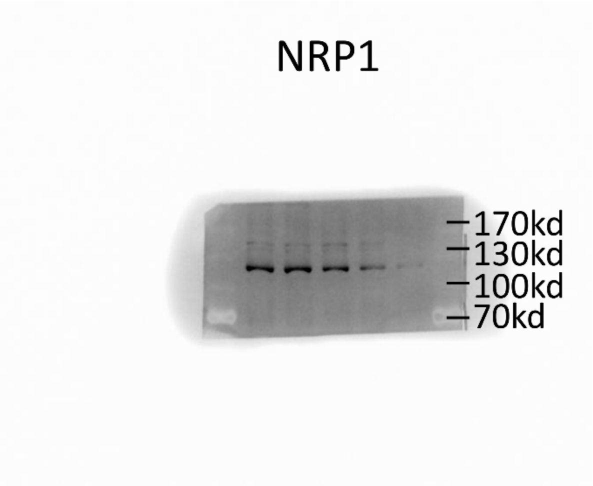

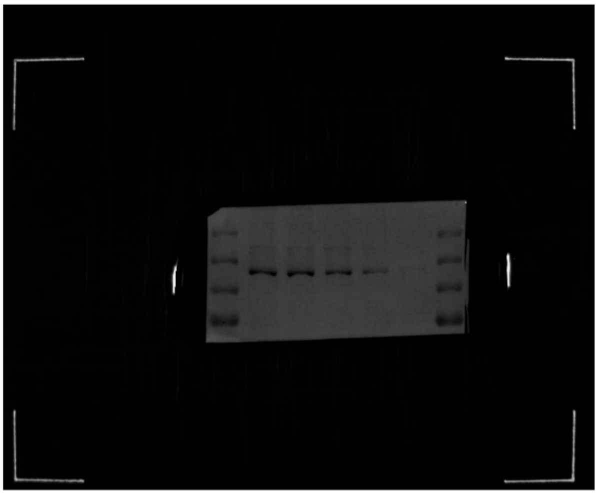

FigS6D

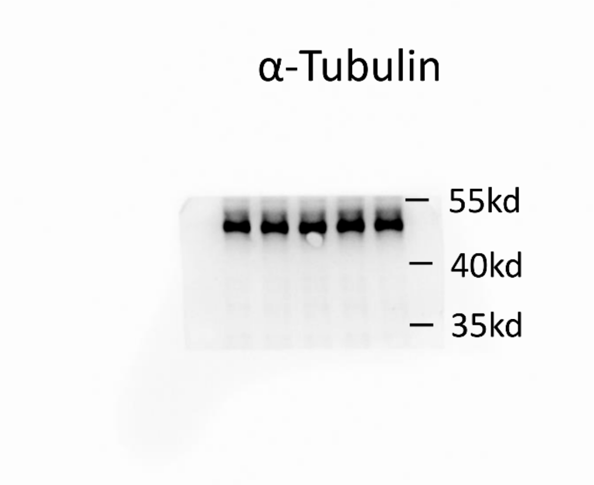

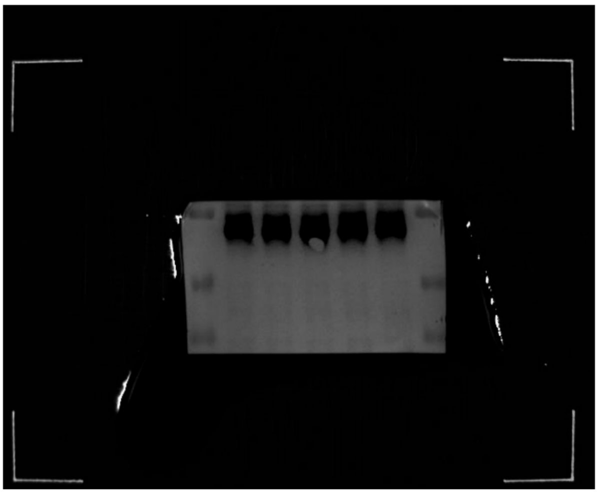

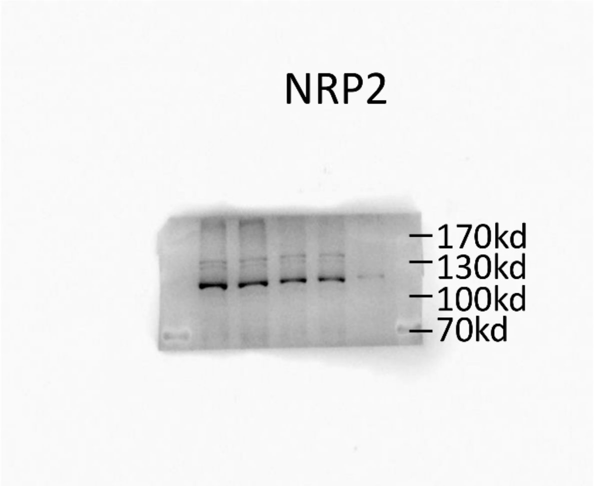

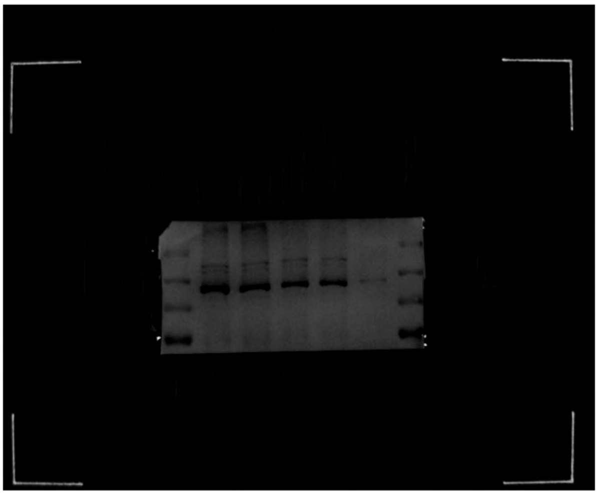

Figure S8. All original images.
